# Unusual Aspects of Charge Regulation in Flexible Weak Polyelectrolytes

**DOI:** 10.3390/polym15122680

**Published:** 2023-06-14

**Authors:** Pablo M. Blanco, Claudio F. Narambuena, Sergio Madurga, Francesc Mas, Josep L. Garcés

**Affiliations:** 1 Physical Chemistry Unit, Materials Science and Physical Chemistry Department & Research Institute of Theoretical and Computational Chemistry (IQTCUB), Barcelona University (UB), 08028 Barcelona, Catalonia, Spain; s.madurga@ub.edu; 2 Grupo de Bionanotecnologia y Sistemas Complejos, Infap-CONICET & Facultad Regional San Rafael, Universidad Tecnológica Nacional, San Rafael 5600, Argentina; claudionarambuena@gmail.com; 3 Chemistry Department, Technical School of Agricultural Engineering & AGROTECNIO, Lleida University (UdL), 25003 Lleida, Catalonia, Spain; joseplluis.garces@udl.cat

**Keywords:** charge regulation, weak polyelectrolyte, acid–base equilibria, adsorption on the wrong side of the isoelectric point, macromolecular crowding, transfer matrix techniques, constant pH simulation, site binding rotational isomeric state model

## Abstract

This article reviews the state of the art of the studies on charge regulation (CR) effects in flexible weak polyelectrolytes (FWPE). The characteristic of FWPE is the strong coupling of ionization and conformational degrees of freedom. After introducing the necessary fundamental concepts, some unconventional aspects of the the physical chemistry of FWPE are discussed. These aspects are: (i) the extension of statistical mechanics techniques to include ionization equilibria and, in particular, the use of the recently proposed Site Binding-Rotational Isomeric State (SBRIS) model, which allows the calculation of ionization and conformational properties on the same foot; (ii) the recent progresses in the inclusion of proton equilibria in computer simulations; (iii) the possibility of mechanically induced CR in the stretching of FWPE; (iv) the non-trivial adsorption of FWPE on ionized surfaces with the same charge sign as the PE (the so-called “wrong side” of the isoelectric point); (v) the influence of macromolecular crowding on CR.

## 1. Introduction

Polyelectrolytes (PE) are polymers containing multiple charged groups which are ubiquitous both in nature and in many of our daily-use products [1]. The electric charge of PEs is usually acquired by means of the dissociation of basic and acidic groups in water, a solvent in which they are typically highly soluble. The physical chemistry of PEs is to a large extent determined by the balance of thermal fluctuations and electrostatic interactions. The resulting behavior, highly system-dependent, has challenged researchers in the field for many years [2].

PEs are frequently classified into strong PEs and weak PEs, in analogy with strong and weak monomeric acids and bases. While strong PEs completely dissociate in solution at any accessible pH, the degree of dissociation of weak PEs is pH-dependent. Consequently, the charge of weak PEs is a fluctuating quantity [3]. Owing to the reversible nature of the acid/base equilibria, weak PEs are able to regulate their charge in response to external perturbations such as electric fields or changes in the solution composition (for example, changes in the pH on solution). The term Charge Regulation (CR) has been coined to refer to this ability of weak PEs. CR plays a fundamental role in a wide range of phenomena such as ligand-receptor interactions in biochemistry [4,5,6,7], drug delivery [8], supramolecular chemistry [9,10,11], binding of metal ions to organic matter in aquatic systems [12,13,14,15,16,17], colloid stability [18], protein–protein and protein–surface interactions [19,20], among many other examples. Due to the importance of CR for many applications, CR has been the target of many recent research studies [21].

Although PEs can adopt rigid or semi-flexible structures, they are in general very flexible molecules [3,22]. In the case of flexible weak PEs, their ionization and conformational degrees of freedom are in general strongly coupled; a change in the ionization state of the PE modifies the electrostatic interactions, forcing the molecule to adopt a different conformation. In the same way, perturbations in their conformational state, causing a change in the distances between charged groups in the chain, can affect the ionization state of the PE [23,24,25,26,27,28,29,30,31]. As a result, new theoretical [22,32,33,34,35,36] and computational approaches [37,38,39,40] have been recently developed. In this review, we present several examples in which CR produces unexpected or non-trivial effects related to the coupling between ionization and chain flexibility.

The review is organized as follows. We introduce basic concepts necessary to understand CR in flexible weak PE in Section 2. In Section 3, we explain the state of the art on theoretical models used to study the ionization of flexible weak PEs. We summarize the current techniques to simulate weak PE at constant pH in Section 4. We discuss how charge regulation affects the elasticity of weak PE in Section 5. We review how charge regulation affects the adsorption of weak PE onto different charged objects in Section 6. In Section 7, we discuss how the presence of macromolecular crowding can affect the properties of weak PE, possibly inducing a charge regulation response. We end the review giving an outlook on current open questions in Section 8.

## 2. Fundamental Concepts on Weak Polyelectrolytes

### 2.1. Ionization Properties

Aiming to facilitate the overall comprehension, we start this review providing a brief summary on the basis of protonation equilibria and electrostatic interactions. This section, together with Section 2.2, contains the fundamental concepts necessary to understand the rest of the review. For a more complete description of the physicochemistry of polyelectrolytes, we invite the interested lecturer to read the extensive books and reviews on the topic [2,41,42,43,44,45,46,47,48,49,50,51]. Lecturers who are already familiar with these topics can consider skipping these sections and directly going to the next sections, where various state-of-the-art applications are reviewed.

#### 2.1.1. Protonation Equilibria

Weak PEs are composed of multiple weak acidic and basic groups or “protonating sites”, which undergo reversible acid/base reactions

(1) $$\begin{array}{cc} \; & \mathrm{HA}⇄A^{-}+H^{+}, \end{array}$$

(2) $$\begin{array}{cc} \; & {\mathrm{HB}}^{+}⇄B+H^{+}, \end{array}$$

where a protonated acid HA or base ${\mathrm{HB}}^{+}$ releases a proton $H^{+}$ to the solution. For a single ligand (in this case, the proton cation), the chemical equilibrium is governed by its corresponding acidity intrinsic acid or dissociation constant

(3) $$K_{a}=\frac{a_{X}a_{H^{+}}}{a_{\mathrm{HX}}}$$

where $a_{H}$, $a_{\mathrm{HX}}$ and $a_{X}$ denote the activities of the proton, the protonated and the unprotonated group. Traditionally, $\mathrm{pH}=-\log_{10}a_{H^{+}}$ and p$K_{a}=-\log_{10}K_{a}$ are expressed in decimal logarithmic units.

In the study of weak PEs, the fundamental experimental quantity is the protonation degree, $\theta =N_{HX}/N$, where $N_{HX}$ is the number of protonated sites and *N* the total number of sites in the macromolecule. θ can be measured by means of potentiometric, spectrophotometric or NMR titrations. NMR titrations also allow the determination of the protonation degree of specific sites of the macromolecule $\theta_{i}$ [52,53,54]. If the ionizable groups are non-interacting and chemically identical, a situation known as “ideal” binding, they can be treated in the same fashion as a monomeric acid or base so that Equation (3) holds and θ fulfills the Henderson–Hasselbalch (HH) equation

(4) $$\theta =\frac{a_{H}/K_{a}}{1+a_{H}/K_{a}}=\frac{10^{-(\mathrm{pH}-pK_{a})}}{1+10^{-(\mathrm{pH}-pK_{a})}}.$$

In the PE literature, it is usual to extend Equation (3) by defining the effective dissociation constant as [12,55]

(5) $$pK_{a}^{\mathrm{eff}}=\mathrm{pH}+\log\left(\frac{\theta }{1-\theta }\right)$$

Although the definition of $pK_{a}^{\mathrm{eff}}$ is formally equivalent to that of a single acidic or basic group (Equation (3)), $K_{a}^{\mathrm{eff}}$ is no longer a constant but a function of the pH-value. Two main reasons explain this fact. First, the ionizable groups are not chemically identical in general (chemical heterogeneity), so those with larger dissociation constants protonate at lower pH-values. Second, the electrostatic interactions with other charged groups in the same molecule modify the affinity for the proton of the unprotonated sites, the so-called PE effect [14,56,57,58]. Despite the underlying chemical complexity, it can be rigorously shown that $K_{a}^{\mathrm{eff}}$ has a clear microscopic interpretation: it represents the average of the dissociation constants of the protonated groups at a given pH-value, which mathematically can be expressed as [12,59,60,61,62]

(6) $$K_{a}^{\mathrm{eff}}\left(\mathrm{pH}\right)=\sum_{i}^{M}K_{a,i}\varpi_{i}\left(\mathrm{pH}\right)\;\;;\;\;\sum_{i}^{M}\varpi_{i}\left(\mathrm{pH}\right)=1$$

where $K_{a,i}$ is the acidity constant of the group *i* and $\varpi_{i}=\theta_{i}/\theta$ is the statistical weight of that constant at a given pH-value. The sum extends over all the *M* distinct types of protonated groups. By “distinct”, we refer not only to the chemical nature of the site itself (carboxyl, phenol, amine, etc.), but also to the chemical environment where the dissociation takes place. For instance, in a linear chain a site with the nearest-neighbor site charged is regarded as “distinct” of the same site but with its nearest-neighbor uncharged. The only condition underlying the decomposition (Equation (6)) is that an ionizable site can bind only one proton. According to this interpretation, $pK_{a}^{\mathrm{eff}}$ is a measure of *cooperativity* of proton binding, since its variation directly quantifies to which extent the affinity for protons increases or decreases with the pH-value. An alternative measure of cooperativity, widely used in biochemistry, is the Hill number [59,63]

(7) $$n_{H}\left(\mathrm{pH}\right)=\frac{d\log\left(\theta /(1-\theta )\right)}{d\mathrm{pH}}$$

In the particular case in which $n_{H}$ is constant, Equation (7) is equivalent to the well-known Hill equation [64]

(8) $$\frac{\theta }{1-\theta }={\left(a_{H}/K_{a}\right)}_{H}^{n}.$$

also known as the Langmuir–Freundlich equation in the context of heterogeneous catalysis chemistry [60,61]. However, in general, $n_{H}$ is not constant but a function of the pH-value. If $n_{H}\left(\mathrm{pH}\right)>1$, it indicates positive cooperativity while $n_{H}\left(\mathrm{pH}\right)<1$ indicates negative cooperativity. Note that both positive and negative cooperativity are possible in the *same* system at different ranges of pH. Nicely, the Hill number has a direct connection to $pK_{a}^{\mathrm{eff}}$ [4,59,63]. By taking derivatives in Equation (5), the following relation holds

(9) $$n_{H}\left(\mathrm{pH}\right)=1-\frac{\mathrm{dp}K_{a}^{\mathrm{eff}}}{d\mathrm{pH}}$$

In the case of ideal binding, $pK_{a}^{\mathrm{eff}}$ is constant, $n_{H}=1$ and the HH equation is recovered. For a negative cooperativity case, as for the majority of PEs, $pK_{a}^{\mathrm{eff}}$ increases with the pH; the derivative in Equation (9) is positive and $n_{H}\left(\mathrm{pH}\right)<1$. Following the same argument, in the case of positive cooperativity, $n_{H}>1$ [4].

Let us consider the ideal situation of non-interacting groups. In this case, the HH equation can be independently applied to each ionizable group

(10) $$\theta_{i}=\frac{z_{i}}{1+z_{i}}\;;\;\theta =\frac{1}{N_{s}}\sum_{i}^{N_{s}}\frac{z_{i}}{1+z_{i}}$$

where $N_{s}$ is the number of sites and $z_{i}=a_{H}/K_{a,i}$ are the so-called reduced site activities, whose associated chemical potential is the *reduced* chemical potential [22]

(11) $$\mu_{i}=-k_{B}T\ln z_{i}=k_{B}T\ln\left(10\right)\left(\mathrm{pH}-pK_{a,i}\right).$$

Note that $\mu_{i}$ depends on the difference between pH and $pK_{a}$, which means that any perturbation affecting $pK_{a}$ is equivalent to a change of the same magnitude in the pH-value [21,65].

In the case of non-interacting sites, the titration curve becomes a superposition of step-like functions according to Equation (10). Each jump occurs at ${\mathrm{pH}}^{\theta_{i}=1/2}=pK_{a,i}$, when the ionizable group is half-protonated. In the literature, this fact has been used to estimate the $pK_{a}$-values of the titrating group directly from the inflection points of the titration curve [66]. However, the method is reliable only if (i) the $pK_{a}$-values are different enough and (ii) electrostatic interactions are weak. Otherwise, Equation (10) is no longer applicable and the obtained $pK_{a}$-values will be shifted with respect to the one corresponding to the isolated group [65].

A very useful observable to quantify the capacity of a weak PE to regulate its charge is the quantity

(12) $$C=-\frac{d\theta }{d\mathrm{pH}}$$

which measures the response of the degree of protonation to changes in the pH-value, in a similar way to how heat capacity quantifies the energy changes when modifying the temperature. *C* has been given many names in the literature such as (protein/charge/differential/ proton/binding) capacitance [6,37,63,67,68,69], charge regulation parameter [70] and charge regulation capacity [71]. Among them, we choose charge regulation capacity to avoid possible confusions with the term “capacitance” used in electrochemistry.

A well-known result of equilibrium statistical mechanics is that response functions are directly related to fluctuations. In particular, the charge regulation capacity C can be expressed in terms of fluctuations of θ as [6,72]

(13) $$C=N_{s}\ln\left(10\right)\left(<\theta^{2}>-<\theta {>}^{2}\right),$$

In other words, the pH-values at which CR is more effective coincide with those at which the fluctuations are maximal. Equation (13) also indicates that *C* is always a positive quantity since the term between brackets is the variance of θ. For non-interacting sites, *C* is obtained by taking derivatives in Equation (10)

(14) $$C=\sum_{i=1}^{N_{s}}\frac{z_{i}}{{\left(1+z_{i}\right)}^{2}}$$

*C* peaks at $z_{i}=1$, i.e., when $\mathrm{pH}=pK_{a,i}$. If the $pK_{a}$ values are separated enough, *C* becomes a succession of peaks [71,73]. In presence of interactions, the peaks are no longer of the form of Equation (14), but they can be wider or narrower and they become asymmetric in general [63,68].

To highlight the importance of *C*, consider the case of a weak PE under an electric field created, for example, by another macromolecule of the solution. As a result, the free energy of the charged groups is shifted by an amount eΔΨ, where *e* is the electron charge and ΔΨ is the increment in the electrostatic potential generated by the applied field. Since the reduced chemical potential depends on the difference $pK_{a,i}-\mathrm{pH}$, the change in the reduced chemical potential can be attributed both to a shift in the acidity constant $pK_{a,i}+e\beta \Delta \Psi$ or, equivalently, to a shift in the pH-value pH−eβΔΨ. However, we know that the maximum response to a change in the pH-value takes place when the C is maximum. Therefore, the maxima of C indicate at which pH-values the system is more sensitive to the external field. The same argument can be applied to any perturbation, different from electric fields, which modifies the protonation free energies [73]. A detailed discussion of the use of *C* in binding and linkage of proteins can be found in Ref. [63]. In short, the pH-values at which the electric field will cause a stronger charge regulation response in the weak PE will be those at which *C* is maximum.

#### 2.1.2. Electrostatic Interactions

Electrostatic interactions are the main driving force which determines the conformational and ionization properties of PE. Consider a system of PEs in a solution with other small ions. Some of these small ions, the *counter-ions*, are released by the PE itself, and preserve the electroneutrality of the PE. The rest of the small ions correspond to other salt ions present in the solution, or *background* electrolytes. The interaction of a pair of charged particles is given by the Coulomb potential

(15) $$U_{C}=\sum_{i=1}^{N_{c}-1}\sum_{j=i+1}^{N_{c}}\frac{1}{4\pi \epsilon_{0}\epsilon_{r}}\frac{q_{i}q_{j}}{r_{ij}},$$

where $N_{c}$ is the number of charged particles; $q_{i}$ and $q_{j}$ are the electric charges of the interacting particles; $r_{ij}$ is the distance between their centers; $\epsilon_{0}$ is the vacuum permittivity and $\epsilon_{r}$ is the relative permittivity of the solvent, which in the case of water at 298 K is $\epsilon_{r}=78.5$. The characteristic length at which electrostatic interactions balance thermal fluctuations, known as the Bjerrum length, can be estimated by equating the coulombic energy between two electron charges to the thermal energy $k_{B}T$

(16) $$l_{B}=e^{2}/\left(4\pi \epsilon_{0}\epsilon_{r}k_{B}T\right).$$

For water at 298 K, $l_{B}\approx 0.71$ nm. In Equations (15) and (16), the solvent is implicitly treated as a continuum dielectric medium; approximation is referred to as *primitive* model by some authors. For solutions of PEs, the primitive model is a more amenable approach for analytical theories and computer simulations than adding explicit water [3,74]. However, there are some applications where solvation effects are important and must be taken into account, for example, in protein chemistry [75].

When there is a sufficient amount of small ions in solution, the electrostatic interactions between charged groups in the PEs are screened by the small ions. The mobile small ions in the solution tend to locate close to the PE charges of opposite charge, decreasing the effective charge of the PE group and ultimately decreasing the resulting electrostatic potential. The most handful way to account for this effect is by using the well-known Debye–Hückel (DH) potential [35,41,76]

(17) $$U_{\mathrm{DH}}=\sum_{i=1}^{N_{P}-1}\sum_{j=i+1}^{N_{P}}\frac{1}{4\pi \epsilon_{0}\epsilon_{r}}\frac{q_{i}q_{j}}{r_{ij}}\exp\left(-r_{ij}\kappa_{D}\right).$$

where $N_{P}$ is the number of charged groups in the PE. It differs from the Coulomb energy (Equation (15)) in the exponential screening term, which becomes important at distances larger than the Debye length $\kappa_{D}^{-1}$

(18) $$\kappa_{D}^{-1}={\left(8\pi IN_{A}l_{B}\right)}^{-1/2}$$

where $N_{A}$ is the Avogadro number and *I* the ionic strength. For water at 298 K, Equation (18) is frequently simplified as

(19) $$\kappa_{D}^{-1}\left(\mathrm{nm}\right)=\frac{0.309}{\sqrt{I(M)}}$$

where $\kappa_{D}^{-1}$ and *I* are expressed in units of nanometer(nm) and molar(M), respectively. For example, for solutions with concentrations of monovalent salt of $10^{-3}$ M, $10^{-2}$ M and $10^{-1}$ mM, the respective Debye lengths are $\kappa_{D}^{-1}\approx$ 9 nm, 3 nm and 1 nm. The sum in (Equation (17)) extends over the $N_{P}$ number of PE charged groups, while the small ions appear only implicitly through the screening term. Using the DH approximation drastically reduces the number of terms to be evaluated, thereby reducing the computer cost for computer simulations [77] and simplifying the development of analytical theories [35,78,79].

Unfortunately, the DH approximation presents some shortcomings. First, it fails when the electrostatic energy exceeds the thermal energy because the linear approximation of the Poisson–Boltzmann equation involved in the derivation of (Equation (17)) breaks down, for example, in the presence of multivalent ions, at high ion concentrations or at high charge densities [80]. Second, the DH potential is only valid if the electrostatic interactions are mediated by the solvent. This is not the case when the charged groups interact through the macromolecular backbone, as happens to neighboring sites in a linear chain, or through a cavity as usual in globular proteins. In these cases, the local dielectric environment is very different from the one in water and more sophisticated forms for the interacting potentials are needed. This task started in the classical works by Kirkwood for a spherical cavity inside a protein [41,81] and was further extended to other geometries [82,83,84].

#### 2.1.3. An Illustrative Example

**Figure 1:**
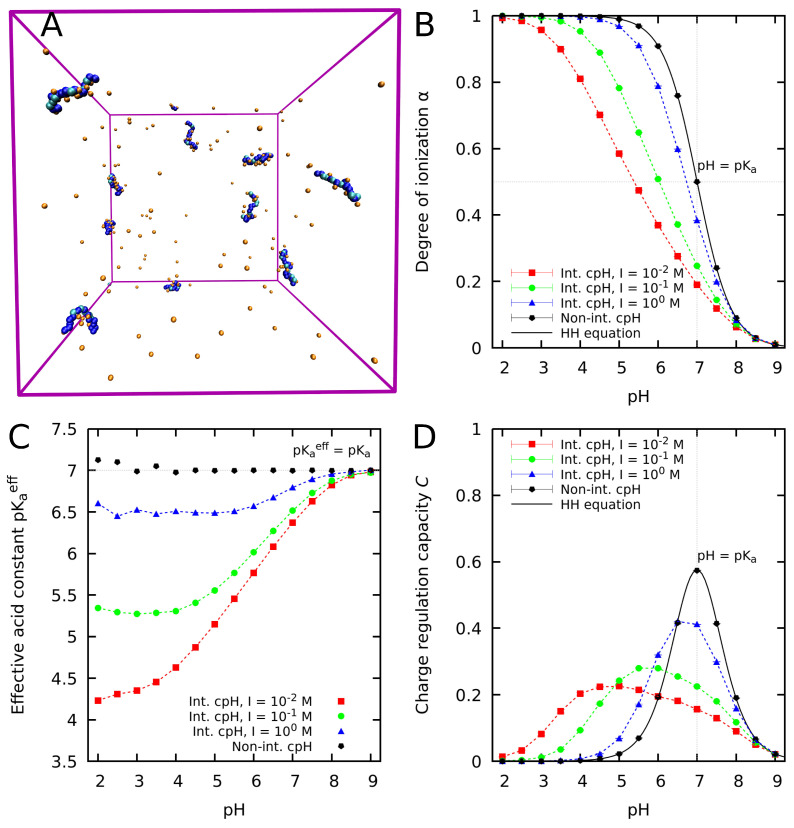
Panel (**A**): Snapshot from a constant pH simulation (Section 4) of a polybase, with $pK_{a}$ = 7, using implicit solvent at pH=6 and ionic strength $I=10^{-1}$ M. The PE is modelled as a set of beads representing the ionizable groups linked by harmonic springs. The color code is: protonated groups (blue), unprotonated groups (cyan) and anionic counter–ions (orange). Other panels: Degree of ionization α (panel (**B**)), effective acidity constant $pK_{a}^{\mathrm{eff}}$ (panel (**C**)) and charge regulation capacity *C* (panel (**D**)) as a function of pH at various *I*–values. Markers correspond to constant–pH simulations while continuous lines are to guide the eyes. The black continuous line corresponds to the Henderson–Hasselbalch (HH) equation.

Let us illustrate the concepts introduced in Section 2.1.1 and Section 2.1.2 with a simple example. In Figure 1, we show a snapshot of a computer simulation of a solution of weak PEs containing 20 basic groups. The macromolecular skeleton is modelled in a coarse-grained fashion as a chain of beads linked by springs. The ionization properties are calculated by means of constant pH Monte Carlo (MC) simulations, a computational technique which will be discussed in Section 4. The solvent is implicit and treated as a continuous dielectric medium (primitive model) while the counter-ions are explicitly considered. The background ionic strength, caused by other salt ions in solution, is included implicitly under the Debye–Hückel approximation (Equation (17)). A simulation snapshot of the simulation is depicted in Figure 1A.

In the PE literature, it is common to study the ionization of PEs in terms of the ionization degree $\alpha =N_{c}/N$, where $N_{c}$ is the number of charged groups and *N* the total number of groups. Note that α is directly related to the degree of protonation θ: for an acid, α=1−θ and for a base, α=θ. Since both quantities yield similar information, the choice between using α or θ is based on the convenience for a particular application. When studying weak PEs, α generally is a convenient choice because it is directly proportional to the amount of charge in the chain for both acid and basic PEs.

In a weak PE, the α-value typically deviates from the ideal result given by the HH equation (Equation (4)) due to the electrostatic repulsion between like-wise charged groups. The titration curve of the PE can be found in Figure 1B, where α as a function of the pH-value is plotted for an ionic strength ranging from $10^{-2}$ M to 1 M. Markers denote simulated points while dashed lines are to guide the lecturer. The black continuous line corresponds to the HH equation (Equation (4)) for which interactions are neglected, usually regarded as the null model in the PE literature [21]. The simulations consistently deviate from this null model towards lower α-values due to the electrostatic repulsion between like-wise charged groups. When no interactions are present in the simulations (black markers), the ideal HH equation is recovered. For a given pH-value, α increases with the ionic strength due to the increase in the screening of the electrostatic repulsion between charged groups. Since this effect stems from the intramolecular interactions within the PE chain, some authors have termed it as the “PE effect” [14,56,57,58].

The effective acidic constant $pK_{a}^{\mathrm{eff}}$ of the PE decreases at low pH-values, where the affinity of the basic groups for proton decreases due to the PE effect. In Figure 1C, this effect can be observed: the effective acidic constant $pK_{a}^{\mathrm{eff}}$ increases as the pH increases until reaching the limiting value of the bare $pK_{a}$ of the basic groups. At high ionic strength, where the PE effect is screened by the salt, the values of $pK_{a}^{\mathrm{eff}}$ are closer to the bare $pK_{a}$ of the basic groups. In the limiting case when interactions are suppressed, $pK_{a}^{\mathrm{eff}}=pK_{a}$ independently of the pH-value.

The peaks of the charge regulation capacity *C* coincide with the inflection points of the titration curve of the PE, marking the pH-values where the PE is more susceptible to charge regulation. In addition, Figure 1D shows that the peaks of the charge regulation capacity *C* shift to lower pH-values and become wider in decreasing the ionic strength. This indicates that at low ionic strength, the PE is susceptible to CR on a broader range of pH-values than at high ionic strength. In the illustrative example presented here, there is only one ionizable group, but in the presence of multiple ionizable groups, *C* can exhibit multiple peaks. In general, *C* is an excellent indicator to pinpoint the pH-values where a given macromolecule is expected to have significant charge regulation effects.

### 2.2. Conformational Properties

In general, macromolecules have a flexible structure that can adopt different spatial arrangements called conformations. Conformations can be inter-converted at room temperature by thermal fluctuations. One should not confuse these conformations with the possible chemical or isomeric configurations arising from the presence of chiral groups. The main difference between conformations and configurations is that to change the configuration of a molecule chemical bonds must be broken, which in general cannot be done at room temperature. Different isomeric configurations exhibit very different physical properties. For homopolymers, the spacial arrangement of the chiral centers of the polymer is referred to as the polymer tacticity [85]. Although tacticity can influence the ionization properties of PEs [86], this topic is out of the scope of this work. In this section, we provide a brief description of the fundamentals of the most basic models which also serves to introduce important concepts to understand the rest of the review but we refer the interested reader to Refs. [87,88] for a more detailed description of these models.

The conformational state of a polymer is given by its conformational degrees of freedom, namely: bond lengths (*l*), bond angles (γ) and dihedral or rotation angles (ϕ) between the planes formed by two consecutive bonds. For flexible linear chains, rotational barriers are usually of the same order of magnitude of the thermal energy $k_{B}T$, so bond rotations constitute the principal mechanism for conformational equilibria at room temperature. Significant changes in the bond lengths and angles are energetically more costly, so usually only small deviations of the averages values are observed. Consequently, a particular conformational state can be well described with a set of rotation angles $\left\{\phi_{i}\right\}$.

While the set of rotation angles defines a conformational state at the microscopic level, the global or macroscopic state is usually measured using either the mean-square end-to-end distance <$r^{2}$> or mean-square radius of gyration $R_{g}^{2}$. <$r^{2}$> is given by

(20) $$<r^{2}>=<{\left(\sum_{i=1}^{N_{b}}\vec{l_{i}}\right)}^{2}>=\sum_{i,j=1}^{N_{b}}<\vec{l_{i}}\cdot \vec{l_{j}}>$$

where $N_{b}$ is the number of bonds in the polymer backbone and $\vec{l_{i}}$ is a vector with the orientation of bond *i* and modulus equal to its bond length $l_{i}$. $R_{g}^{2}$ is defined as the mean-square distance of the monomers to average position ${\vec{r}}_{\mathrm{av}}$

(21) $$R_{g}^{2}=\frac{1}{N_{b}+1}<\sum_{i=1}^{N_{b}+1}{\left(\vec{r_{i}}-{\vec{r}}_{\mathrm{av}}\right)}^{2}>=\frac{1}{2{\left(N_{b}+1\right)}^{2}}\sum_{i,j=1}^{N_{b}+1}<r_{ij}^{2}>$$

where ${\vec{r}}_{i}$ is the position of monomer *i* and $r_{ij}$ is the distance between the monomers *i* and *j*. The second identity in Equation (21) is due to Lagrange [87].

#### 2.2.1. Basic Models

In many cases, it is not necessary to introduce the full conformational details to develop a theory able to explain experimental observations. Frequently, it is enough for the theory to capture the key conformational features of the particular system of study. For this reason, many theories build upon simplified or basic models.

The most famous and historically important basic model is the Freely Jointed Chain (FJC), a linear chain with $N_{b}$ bonds randomly articulated. In this model, the orientations of the bond vectors $\vec{l}$ are statistically independent, so that $<\vec{l_{i}}\cdot \vec{l_{j}}>=0$ for any pair of different bonds i≠j. For a long enough FJC composed of bonds with the same length, the square end-to-end distance reads [87]

(22) $$<r^{2}>=N_{b}l^{2}.$$

It follows from Equation (22) that the end-to-end distance of a FJC chain increases much more slowly with $N_{b}$ than its contour length $L_{C}=N_{b}l$. This result is a natural consequence of the random coil nature of the FJC, which represents the qualitative behavior of many linear polymer chains.

The bond orientations in polymer chains are not statistically independent, which breaks the main assumption of the FJC model. Nevertheless, bond orientations become uncorrelated after a characteristic distance known as the *persistence length* $l_{p}$. For a long enough chain, $l_{p}$ can be mathematically defined as

(23) $$l_{p}=\frac{1}{l}<\sum_{j=i+1}^{N_{b}}\vec{l_{i}}\cdot \vec{l_{j}}>$$

which is the sum of the projections of the bonds following a given bond along the backbone of the polymer chain. Note that Equation (23) implies that the bonds are identical. Otherwise, one should define a persistence length for each kind of bond.

Let us show that, if correlations decay fast enough along the chain, $<r^{2}>$ is proportional to $N_{b}$ for any polymer chain by replacing Equation (23) in Equation (20)

(24) $$<r^{2}>=N_{b}\left(2l_{p}+1\right)l=N_{b}l_{K}l$$

where $l_{K}=2l_{p}+l$ is the Kuhn length [89,90,91]. Indeed, in such a case, $<r^{2}>$ is still proportional to $N_{b}$ in a similar fashion as in the FJC model (Equation (22)). Chains with this property are known as gaussian chains. This is a key result, since it is a corner stone for further theoretical derivations. For example, for a long-enough gaussian chain, the mean square radius of gyration is one sixth of the mean-square end-to-end distance [85].

Let us elaborate our theoretical considerations by assuming that the bending energy of two consecutive bonds only depends on their bond angle γ. Then, one can prove that the correlation between any two bonds *i* and *j* is equal to [85]

(25) $$<\vec{l_{i}}\cdot \vec{l_{j}}>=\tau^{\left(j-i\right)}$$

where τ=<cos(π−γ)> is the average cosine of the bond angle. Replacing Equation (25) in Equation (23), the corresponding persistence length reads

(26) $$l_{p}=\frac{\tau }{1-\tau }l.$$

Two limiting situations can be considered as particular cases of Equation (26). If the bending angle is constrained to adopt only one value, we obtain the Freely Rotating Chain (FRC). On the other hand, one can consider the case in which the chain can be treated as a continuous curve. In this case, *l* tends to zero while τ tends to one in such a way that the persistent length (26) remains finite. The resulting model is the very popular worm-like chain (WLC) or Kratky–Porod model [92,93].

Both FJC and WLC are gaussian chains which do not account for the long-range correlations created by long-range and excluded volume interactions, which are the defining features of the so-called *self-avoiding* chains. Flory proved that, for a self-avoiding chain, the mean square end-to-end distance scales with $N_{b}$ as

(27) $$<r^{2}>\;\propto N_{b}^{2\nu }$$

with ν≈3/5, which is faster than predicted for a gaussian chain (Equation (22)) [94]. The scaling law (27) also holds for highly charged PEs [31], in which repulsive electrostatic interactions cause a self-avoiding behavior.

The FJC and WLC models can be understood as *coarse-grained* representations of the polymer, in the sense that the details of the microscopic structure of the polymer are reduced to a few effective parameters. However, more advanced statistical mechanics techniques can be used to derive expressions for the global conformational quantities (persistent length, end-to-end distance) in terms of microscopic properties (rotational energies, bond lengths and angles).

#### 2.2.2. The Rotational Isomeric State (RIS) Model

The Rotational Isomeric State (RIS) model was developed by Flory in his important book “Statistical Mechanics of Chain Molecules” [85]. Despite the fact that the classic theory was developed in the 1960s, it is still a guide to polymer research, as shown in a recent book of collected chapters [95]. The two approximations involved in the RIS model are: (i) the values of the bond lengths and bond angles can be considered constant and (ii) the number of rotational states is finite. Both approximations constitute the basis of the RIS model and they are justified as follows.

Assumption (i) has already been discussed at the beginning of this Section 2.2: Bond angles and bond lengths are rather still degrees of freedom while rotation barriers usually are of the order of thermal energy at room temperature; therefore, one can assume that the set of rotation angles ϕ essentially determines to a large extent the conformational state of linear chains [85]. Although ϕ can adopt any value from 0 to 2π in principle, only those corresponding to energy minima are significantly populated (typically, trans (*t*), gauge+ ($g^{+}$) and gauge− ($g^{-}$ states). Assumption (ii) builds on this argument to reduce the continuum of rotational states to a finite number. Both approximations together permit a microscopic description of the conformational properties of flexible polymers that is amenable to statistical mechanics.

Within these assumptions, the free energy can be written as a sum over contributions of the rotational states

(28) $$F_{\mathrm{RIS}}\left(\left\{\phi \right\}\right)=\sum_{i=1}^{N_{R}}F_{1}\left(\phi_{i}\right)+\sum_{i=1}^{N_{R}-1}F_{2}\left(\phi_{i},\phi_{i+1}\right)+\cdots$$

where $N_{R}$ is the number of rotating bonds (i.e., those with an associated ϕ), $F_{1}\left(\phi_{i}\right)$ represents the intrinsic rotational energy associated to a rotating bond *i* and $F_{2}\left(\phi_{i},\phi_{i+1}\right)$ is the pair interaction energy between two consecutive rotating bonds. Multiplet interactions between three or more rotating bonds can also be included, but nearest-neighbor interactions are often enough to describe many properties of interest. It follows that the probability of a conformational state is given by

(29) $$P\left(\left\{\phi \right\}\right)=\frac{\exp(-\beta F(\{\phi \}))}{Z_{\mathrm{RIS}}},$$

where $Z_{\mathrm{RIS}}$ is the normalization constant or RIS canonical partition function

(30) $$Z_{\mathrm{RIS}}=\sum_{\left\{\phi \right\}}\exp\left(-\beta F\left(\left\{\phi \right\}\right)\right)=\sum_{\left\{\phi \right\}}\prod_{i=2}^{N_{R}}\sigma_{i}\;\Psi_{i}$$

where $\sigma_{i}=\exp\left(F_{1}\left(\phi_{i}\right)\right)$ and $\Psi_{i}=\exp\left(F_{2}\left(\phi_{i},\phi_{i+1}\right)\right)$ are the Boltzmann factors associated to the rotational energy of bond *i* and to its pair interaction with the neighboring rotating bond i+1, respectively. $Z_{\mathrm{RIS}}$ contains all the information about the conformational properties of the polymer.

Since the number of conformational states grows as $3^{N_{R}}$, P({ϕ}), the properties derived from it can only be calculated by direct enumeration of states for short polymers. Otherwise, computer simulations or statistical mechanics techniques must be used. In the case of linear molecules, $Z_{\mathrm{RIS}}$ can be evaluated by means of the Transfer Matrix (TM) method, a technique borrowed from statistical mechanics [96] and the fundamental tool of the RIS model. The basic idea of the TM technique is to relate the partition function of a polymer with $N_{R}+1$ rotating bonds with that of a polymer with $N_{R}$ rotating bonds. To do so, we need to multiply and sum the new Boltzmann factors. The TM method permits to express this relationship in a linear form, ultimately allowing to calculate the $Z_{\mathrm{RIS}}$ of a polymer with any chain length following a recursive strategy.

**Figure 2:**
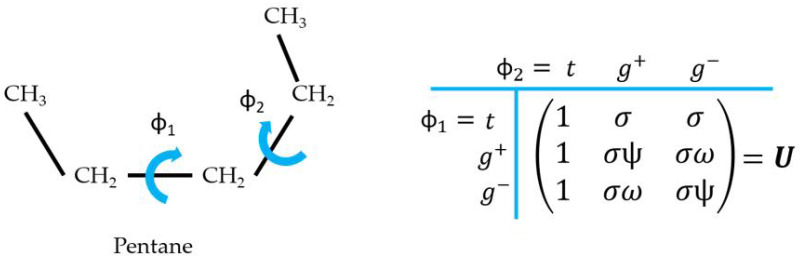
Scheme linking the chemical structure of penthene with the corresponding transfer matrix used to solve its Rotational Isomeric State model. Figure reprinted with permission from the PhD. thesis of Pablo M. Blanco [97].

As a simple example, we review the basics of the RIS model and the TM method considering the case of the penthane molecule. Penthane only has two rotation angles (i.e., two rotating bonds), as illustrated in the scheme in Figure 2. Using the RIS approach, we assume that these two rotation angles $\phi_{1}$ and $\phi_{2}$ can only adopt the rotational states of minimum energy: *t*, $g^{+}$ and $g^{-}$. To design the TM of penthane, U, we use the following key idea: each row of U corresponds to a different conformational state of the *first* rotating bond while each column of U corresponds to a different conformational state of the *second* rotating bond, as outlined in Figure 2. Therefore, each element $U_{ij}$ matches a unique combination of $\phi_{1}$ and $\phi_{2}$. The resulting TM reads

(31) $$U=\left(\begin{array}{ccc} 1 & \sigma & \sigma \\ 1 & \sigma \omega & \sigma \psi \\ 1 & \sigma \psi & \sigma \omega \end{array}\right).$$

where we have chosen the origin of energy as the conformation with all bonds in *t*. Thus, $\sigma_{t}=\exp\left(-\beta F_{1}\left(t\right)\right)=1$ and all the elements of U matching $\phi_{2}=t$ equate to 1. Following the same logic, the other elements of U include in their Boltzmann factor the energy of adding a bond in a gauche conformation $\sigma =\exp(-\beta E_{1}\left(g\right))$. Note that the intrinsic rotational energy of $g^{+}$ and $g^{-}$ states is the same due to the symmetry of penthane. Also owing to symmetry, penthane has only two pair interaction energies corresponding to Boltzmann factors: $\omega =\exp\left(-\beta E_{2}\left(g^{+},g^{+}\right)\right)=\exp\left(-\beta E_{2}\left(g^{-},g^{-}\right)\right)$ and $\psi =\exp\left(-\beta E_{2}\left(g^{+},g^{-}\right)\right)=\exp\left(-\beta E_{2}\left(g^{-},g^{+}\right)\right)$. Once U is properly defined, the TM machinery can be used to calculate $Z_{\mathrm{RIS}}$ as a matrix product

(32) $$Z_{\mathrm{RIS}}\left(\mathrm{penthane}\right)=p\;U^{2}\;q^{T},$$

where p=(1,0,0) and q=(1,1,1) are the initial and final vectors, whose function is to perform the sums over the three possible states for the first and the last rotating bond, respectively.

It is straightforward to extend the method when the bonds can adopt more than three possible states by adding rows and columns to the TMs. Moreover, bonds do not need to be identical since different TMs can be assigned to each bond to study linear chains of any arbitrary composition. In general, the $Z_{\mathrm{RIS}}$ of any linear polymer can be calculated using the TM method as

(33) $$Z_{\mathrm{RIS}}=p\;\prod_{i=1}^{N_{R}}U_{i}\;q^{T}.$$

Statistical mechanics can be used to calculate many conformational properties from $Z_{\mathrm{RIS}}$. For example, the probability that a bond *j* is in a gauche state (assuming a chain with symmetry such that $g^{+}=g^{-}$) reads

(34) $$P_{j}\left(g\right)=-k_{B}T\frac{\partial \ln Z_{\mathrm{RIS}}}{\partial \ln\sigma_{j}}.$$

Similar expressions can also be derived to measure other more common conformational quantities such as the end-to-end distance or the radius of gyration. For brevity, we write here the expression for the radius of gyration and we refer to Ref. [85] for the equivalent expressions for the end-to-end distance. Within the RIS model, the radius of gyration can be calculated using the TM machinery as

(35) $$R_{g}^{2}=\frac{2}{N_{R}Z_{\mathrm{RIS}}}J\prod_{i=1}^{N_{R}}S_{i}J^{\prime }$$

where the supermatrix $S_{i}$ reads [85]

(36) $$S_{i}={\left(\begin{array}{ccccc} U & U & \left(U⊗l^{T}\right)∥T∥ & \left(l^{2}/2\right)U & \left(l^{2}/2\right)U\\ 0 & U & \left(U⊗l^{T}\right)∥T∥ & \left(l^{2}/2\right)U & \left(l^{2}/2\right)U\\ 0 & 0 & \left(U⊗E\right)∥T∥ & U⊗l & U⊗l\\ 0 & 0 & 0 & U & U\\ 0 & 0 & 0 & 0 & U \end{array}\right)}_{i}$$

where ⊗ is the matrix direct product, *l* is the bond length, $l^{T}=\left(1,0,\;\ldots ,\;0\right)$, E and 0 are the identity and zero matrices, respectively, and

(37) $$\begin{array}{c} J=\left(1,1,1,\;\ldots ,\;0,0,0\right)\\ J^{\prime }={\left(1,1,1,\;\ldots ,\;1,1,1\right)}^{T}. \end{array}$$

Note that the dimensions of $l^{T}$, E, 0, J, $J^{\prime }$, ${∥T∥}_{i}$ need to be properly adjusted to match the dimensions of U, which in turn depend on the number of rotational states considered. For all the arrays except for ${∥T∥}_{i}$, one only needs to add more 0 s or 1 s into the array to adapt it to the dimensions of U following the recipe given by Flory [85]. For ${∥T∥}_{i}$, one needs to define a super matrix using one rotation matrix per each rotational state. For example, in the case that only *t*, $g^{+}$ and $g^{-}$ states are considered, ${∥T∥}_{i}$ reads

(38) $${∥T∥}_{i}={\left(\begin{array}{ccc} T\left(t\right) & 0 & 0\\ 0 & T\left(g^{+}\right) & 0\\ 0 & 0 & T\left(g^{-}\right) \end{array}\right)}_{i},$$

with the rotation matrix given by

(39) $$T_{i}\left(\phi \right)={\left(\begin{array}{ccc} \cos\left(\pi -\gamma \right) & \sin\left(\pi -\gamma \right) & 0\\ \sin\left(\pi -\gamma \right)\cos\left(\phi \right) & -\cos\left(\pi -\gamma \right)\cos\left(\phi \right) & \sin\left(\phi \right)\\ \sin\left(\pi -\gamma \right)\sin\left(\phi \right) & -\cos\left(\pi -\gamma \right)\sin\left(\phi \right) & \cos\left(\phi \right) \end{array}\right)}_{i}.$$

The sub-index *i* in Equations (35)–(39) indicates that the matrix U and the bond lengths are in general different for each bond *i*.

#### 2.2.3. Elasticity

Understanding the mechanical response of a single polyelectrolyte chain to an external force is the first step to tackle many problems in polymer science such as gel swelling, contraction of bio-fibrils (e.g., myofibrils) or the behavior of elastomers such as rubber. Recent advances in experimental techniques for single-molecule manipulation, such as Atomic Force Microscopy (AFM) and optical tweezers [98], have allowed direct investigation of processes involving mechanical stretching such as DNA elasticity [99,100,101,102], mechanically-induced chemical reactions [103,104,105] and conformational transitions [106], force-dependent enzyme cathalisis [107], and protein unfolding [108] and desorption [109], among many others [110]. Furthermore, advanced techniques have allowed direct investigation of the mechanical properties in high vacuum, at high pulling speed and along different force directions [111,112,113,114,115].

Before the technology for performing single-molecule experiments was available, the field was restricted to only theoretical considerations [116]. The starting point for many of these theories consists of adding a term of mechanical work to the conformational free energy of the models described in the previous sub-sections

(40) $$F_{\mathrm{str}}\left(\left\{\phi \right\}\right)=E\left(\left\{\phi_{i}\right\}\right)+\left(\sum_{i=1}^{N_{b}}\vec{l_{i}}\right)\cdot \vec{F}$$

where $\vec{F}$ is the applied force and $E\left(\left\{\phi \right\}\right)$ is the energy at zero applied force.

Usually, basic models introduced in Section 2.2 are chosen to define $E\left(\left\{\phi_{i}\right\}\right)$ in Equation (40). With the help of statistical mechanics, expressions for the extension of the polymer in the direction of the pulling force $L_{z}$ as a function of the applied force can be derived. For a Freely Jointed Chain (FJC), $L_{z}$ reads [117,118]

(41) $$L_{z}\left(\mathrm{FJC}\right)=L_{c}\left(\coth\left(\beta Fl_{k}\right)-\frac{1}{\beta Fl_{k}}\right),$$

were $l_{k}$ is the Kuhn length, $L_{c}$ is the contour length of the polymer. Marko and Siggia proposed an approximate force-extension curve for the worm-like chain (WLC) [119,120]. Many authors have proposed modifications of the FJC and WLC models, including deformation of bond lengths or angles [102,121,122] and freely rotating bonds [35]. An exhaustive compilation of single-chain stretching experiments which are interpreted in terms of FJC and WLC models, including synthetic polymers, dendrimers and polysaccharides, among other biopolymers, can be found in Ref. [110].

Alternatively, first-principles methods accounting for the microscopic details of the molecule have been proposed. By means of quantum calculations, the most stable conformational states of a small number of interacting monomers are selected. Then, MC simulations or transfer matrix methods are used to obtain the conformational properties. The resulting scheme can be regarded as an adaptation of the RIS model to the stretching problem. This approach has been successfully applied to poly-ethylene glycol (PEG) [123,124,125,126] or to poly-peptides [127,128].

Macromolecular stretching proceeds in a *hierarchical* manner so that throughout the stretching process, degrees of freedom of increasing energy are activated, resulting in different *force regimes* [116]. At very low forces (F≲1 pN), the response to the external force of chain is due to the entropy loss in elongating the chain. The chain thus behaves as an *entropic spring* and the extension fulfills the linear law

(42) $$\frac{L_{z}}{N_{b}l}=\frac{l_{K}F}{3k_{B}T}$$

which is valid regardless of the polymer chemical structure. In the presence of long-range correlations generated by excluded volume effects, this makes $L_{z}$ rapidly deviate from Equation (42). In this case, Pincus proposed the scaling law [129]

(43) $$L_{z}\propto F^{1/\nu -1}.$$

with ν=3/5. Note that for ν=1/2, the linear law (Equation (42)) is recovered. As the force increases, the rotational, bending and stretching degrees of freedom are successively activated [29]. Moreover, as force increases, microscopic chain deformations act collectively, microscopic details are irrelevant and coarse-grained models become a good approximation. Furthermore, these classic FJC and WLC models can be modified to include the enthalpic elasticity calculated by Quantum Mechanics (QM) computations [130,131]. These QM-modified models have been found to fit well the force-extension of polymers with different side chains in conditions where the specific interactions of such groups can be neglected [132,133]. However, more refined models including kinetic effects such as the the two-state QM are necessary when the side groups of the polymer have a significant interaction with the solvent [134]. Note that so far ionization degrees of freedom have not been included in the theoretical treatment. This issue will be discussed in Section 5.

## 3. Theoretical Models for Weak Polyelectrolytes

### 3.1. The Site Binding (SB) Model

One of the most used models in statistical mechanics is the Ising model, which has been the starting point for the theoretical treatment of magnets and gas adsorption [64,96]. The Ising model describes the system as a set of units (for example, spins or sites) which can adopt two possible states. When this approach is applied to the binding of proton to weak PEs, one obtains the Site Binding (SB) model [83]. The reason for such renaming is that the SB model assumes a localized binding of proton to one group or binding site. In other words, there is a one-to-one association between bound protons and binding sites. Although delocalized proton binding has been reported in certain cases [53,135], in which the proton can be shared by different chemical groups, in general it is rather established that proton binding is localized.

Within the SB model, a particular protonation state or *microstate* [3] is characterized by a set of variables $s=\left\{s_{i}\right\}$, where $s_{i}=1$ if the site *i* is protonated and $s_{i}=0$ otherwise. The free energy of a microstate can be expressed as the so-called cluster expansion [3,22]

(44) $$F_{\mathrm{SB}}\left(s\right)=\sum_{i}^{N_{s}}\mu_{i}s_{i}+\sum_{i>j}^{N_{s}}\epsilon_{ij}s_{i}s_{j}+\sum_{i>j>k}^{N_{s}}\tau_{ijk}s_{i}s_{j}s_{k}+\ldots ,$$

where $N_{s}$ is the number of protonating sites, $\mu_{i}$ is the reduced chemical potential of the site *i* introduced in Section 2.1.1, $\epsilon_{ij}$ is the interaction free energy between sites *i* and *j* and $\tau_{ijk}$ is the three-body or triplet interactions, and so on. In many cases, the cluster expansion converges very quickly to the exact free energy. By using the symmetries of the molecule, the number of parameters in Equation (44) can be drastically reduced [34]. Note that in Equation (44), we assume that the PE is a polybase; therefore, a ’deprotonated’ state corresponds to the ’uncharged’ state. However, the main ideas can be readily extended to polyacids and polyampholytes [32]. Moreover, by allowing the state variables to adopt more than two values, the formalism can be extended to account for competitive binding of metal ions [36].

The probability of a particular microstate is given by

(45) $$P\left(s\right)=\frac{e^{-\beta F_{\mathrm{SB}}\left(s\right)}}{\Xi_{\mathrm{SB}}}$$

where $\Xi_{\mathrm{SB}}=\sum_{s}e^{-\beta F_{\mathrm{SB}}\left(s\right)}$ is the SB semi-grand canonical partition function, which contains all the information about the ionization properties. For instance, the degree of protonation of site *i*, $\theta_{i}$, can be expressed as

(46) $$\theta_{i}=-k_{B}T\frac{\partial \ln\Xi_{\mathrm{SB}}}{\partial \mu_{i}}.$$

When the number of sites is small, $\Xi_{\mathrm{SB}}$ can be calculated by direct enumeration [34]. This approach becomes unfeasible for a large number of sites (N>20), since the number of microstates grows exponentially with *N*. In this case, computer simulations or transfer matrix techniques borrowed from Statistical Mechanics become necessary. Transfer matrix techniques are especially well suited for systems dominated by short-range interactions. However, the transfer matrix formalism can also be extended to include long-range interactions in an effective way. Let us review the transfer matrix formalism first for systems with only short-range interactions and later we will show how to extend this formalism to include long-range interactions.

#### 3.1.1. Short-Range (SR) Interactions

One can analytically calculate $\Xi_{\mathrm{SB}}$ for linear chains when only short-range interactions are considered using the transfer matrix method. Analogously to what we reviewed for the RIS model in Section 2.2.2, the method consists of relating recursively the partition function corresponding to a system with $N_{s}+1$ sites to the partition function of the one with $N_{s}$ sites. Again, the relationship is linear and it can be expressed in terms of transfer matrix.

Let us showcase the transfer matrix method applied to the SB model for the simple case: a linear polybase of identical sites with only nearest-neighbor interactions of energy equal to ϵ. For convenience, we take the origin of energy as the state when the PE is fully deprotonated. In this case, the transfer matrix T is designed as follows: each *row* corresponds to a different protonation state of the *previous* binding site and each *column* corresponds to a different protonation state of the *next* binding site of the PE. In this case, T reads

(47) $$T=\left(\begin{array}{cc} 1 & z\\ 1 & zu \end{array}\right)$$

where z=exp(−βμ) is the reduced activity of proton and u=exp(−βϵ) is the Boltzmann factor corresponding to the interaction between two protonated groups. The energy origin is taken as the fully deprotonated molecule. Note that the first column contains ones since it corresponds to adding a non-protonated empty site, which equates to a Boltzmann factor of 1 due to our choice of the origin of energy. In the second column, a protonated site is added to a deprotonated site (first row) and to a protonated site (second row) which implies adding a Boltzmann factor of *z*. In the latter case, an additional Boltzmann factor of *u* needs to be added to account for the interaction between the two protonated sites. Once T is built, one can calculate the SB with the product

(48) $$\Xi_{\mathrm{SB}}=pT^{N}q^{\prime }$$

with initial and final vectors p=(1,0) and q=(1,1).

The transfer matrix technique is very versatile and can be readily extended to include next-nearest-neighbor interactions [136], triplet interactions [137], competitive metal binding [36] or polyampholytes [22,138]. However, since the size of the transfer matrices grows exponentially with the number of neighboring sites [139], the method is in principle restricted to short-range interactions. However, this approach can be extended to include long-range interactions in an approximate but very accurate way using the Local Effective Interaction Parameters (LEIP) method [79,140].

#### 3.1.2. Long-Range (LR) Interactions: Local Effective Interaction Parameters (LEIP)

Long-range interactions are unavoidable in the theoretical description of many systems, for example, solutions at intermediate and low ionic strengths. The standard transfer matrix formalism, only including short-range interactions, is not sufficient to describe such systems. However, a systematic method has been recently developed to include long-range interactions in the transfer matrix formalism in an approximate but very accurate way. The main idea is to replace the original SB free energy (Equation (44)) by a new one involving only SR interactions which account for the LR interactions in an effective way. We refer to this approach as the Local Effective Interaction Parameters (LEIP) method [79].

Let us consider the most simple situation of a chain with identical sites $\mu_{i}=\mu$. The SB free energy (Equation (44)) can be rewritten as the sum $F_{\mathrm{SB}}\left(s\right)=F_{\mathrm{LEIP}}\left(x\right)+\Delta F\left(x\right)$, where

(49) $$\begin{array}{c} F_{\mathrm{LEIP}}\left(x\right)=\left(\mu -x\right)\sum_{i}s_{i}+\epsilon \sum_{i}s_{i}s_{i+1}\\ \Delta F\left(x\right)=\sum_{j>i+1}\epsilon_{ij}s_{i}s_{j}+x\sum_{i}s_{i} \end{array}$$

where *x* represents the correction to the chemical potential due to the presence of LR interactions. Note that the free energy of the unperturbed system $F_{\mathrm{LEIP}}$ corresponds to a system with only nearest-neighbor interactions and effective chemical potential $\mu^{\prime }=\mu -x$, which can be evaluated by means of transfer matrices. The value of *x* is evaluated by using the Gibbs variational principle [96]

(50) $$\Omega \leq \tilde{\Omega }=\Omega_{0}\left(x\right)+{\langle \Delta F\left(x\right)\rangle }_{0}$$

where $\Omega =-k_{B}T\ln\Xi$ is the free energy and $\Omega_{0}\left(x\right)=-k_{B}T\ln\Xi_{0}$ is the free energy of the unperturbed system. ${\langle \cdots \rangle }_{0}$ represents the thermal average of the LR free energy taken over the unperturbed system. As a consequence of the inequality (Equation (50)), the optimal value of *x* is obtained by minimizing $\tilde{\Omega }$. *x* has a clear physical interpretation: it is the average variation in the LR free energy when a site is protonated [79]. The LEIP method can be extended to correct higher-order cluster parameters, such as the nearest-neighbor interactions. However, we have not observed any improvement when adding second-order corrections. This fact indicates that one can replace the PE with LR interactions by an equivalent one with only SR interactions with a corrected reduced chemical potential with great accuracy, at least for rigid systems.

**Figure 3:**
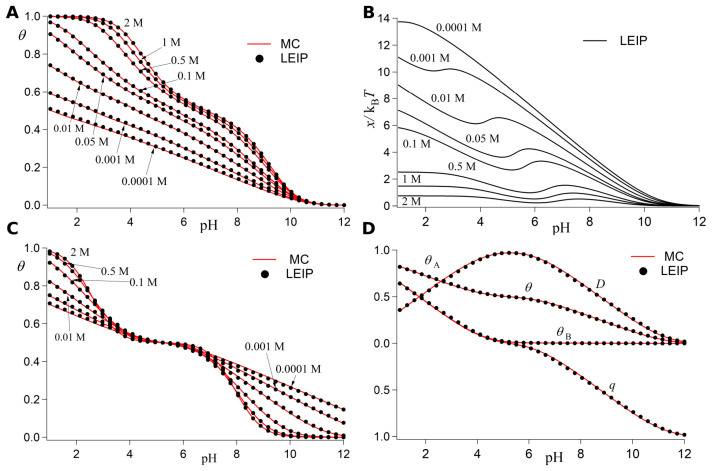
Protonation properties of a model polybase and a model polyampholyte as a function of pH calculated with the LEIP method (red lines) and constant pH Monte Carlo simulation (black markers) at different ionic strength *I* values. Panel (**A**): titration curve of a linear polybase with p$K_{A}=9$. Panel (**B**): LEIP correction to the reduced chemical potential $x^{\prime }$ for the same polybase. Panel (**C**): titration curve of a linear polyampholyte with alternating acidic and basic groups with p$K_{A}$ = 4.5, p$K_{A}=6$, respectively. Panel (**D**): For the same polyampholyte, degrees of protonation of the acidic ($\theta_{A}$) and basic ($\theta_{B}$) groups, average number of zwitterions per monomer (*D*) and average charge per monomer (*q*) at I=0.01 M. Further technical details and parameters of the model can be consulted in Ref. [79]. In all cases, the LEIP method reproduces the cpH calculations within the simulation error. Figures are reproduced with permission from Ref. [79] (Copyright (2017) from Wiley Periodicals LLC).

In Figure 3, we showcase the LEIP method for two different kinds of linear PE: a polybase (upper panels) and a polyampholyte (lower panels) with acidic and basic monomers ordered in an alternating fashion. In all the plots shown in Figure 3, the results given by the LEIP (red lines) are compared with the ones given by constant pH Monte Carlo (cpH) simulation (black dots) where the same system is solved without the approximations involved in the LEIP method. The features of cpH simulation are introduced later in Section 4. To make the theoretical treatment more amenable, the DH potential (Equation (17)) is used in the full range of ionic strength *I*, from $I=10^{-4}$ M to 2 M. The coincidence between the LEIP and the cpH simulation is excellent in all cases, within the numerical error of the computer simulations.

Note that the LEIP method reproduces very well the titration curves in the full range of ionic strength not only for the case of the polybase (Figure 3A) but also in the case of the polyampholyte (Figure 3C,D). In the polybase case, one only needs one correction to the reduced chemical potential $x^{\prime }$, which increases monotonically with the *I*-value, as can be observed in Figure 3B. The polyampholyte case, however, is more complex because both positive and negative charges are present involving both repulsive and attractive electrostatic interactions. Moreover, since the acidic and basic groups are ordered in an alternating fashion, the presence of negative and positive charges is highly correlated so that zwitterions are formed. Mean field theories usually fail to describe the titration curve of zwitterions due to the presence of such correlations. Within the LEIP framework, two corrections to the reduced chemical potentials are needed, those of the acid $x_{A}$ and the base $x_{B}$. Even in this complex case, the LEIP method reproduces with extreme accuracy the protonation properties of the polyampholyte predicted by the cpH simulations. Such protonation properties include not only the protonation degree of the polyampholyte θ (Figure 3C) but also the individual protonation properties (Figure 3D), including: the degrees of protonation of the acidic $\theta_{A}$ and basic $\theta_{B}$ groups, the number of zwitterions per monomer *D* and the total charge *q* per monomer. Overall, the LEIP method is able to calculate very well the protonation properties of both homogeneous and heterogeneous PEs in the full range of *I*-values.

### 3.2. The Site Binding Rotational Isomeric State (SBRIS) Model

So far, the theoretical treatment for the protonation degrees of freedom, presented under the Site Binding (SB) model formalism, was restricted for rigid structures. There are many interesting systems in which charge regulation phenomena take place in rigid structures such as nanoparticles or surfaces [3,79]. However, PE are in general flexible molecules whose conformational and ionization degrees of freedom are strongly coupled [19,31,71,139]. Under the SB formalism, this means that the cluster parameters in Equation (44) become dependent on the conformational state. The number of conformation states is in principle infinite but they can be reduced to a discrete number following the strategy of the Rotational Isomeric State (RIS) model [141], introduced in Section 2.2.2.

The result of combining the SB model with the RIS model formalism is the recently proposed Site Binding Rotational Isomeric State (SBRIS) model [25,79]. The SBRIS free energy reads

(51) $$F_{\mathrm{SBRIS}}\left(s,c\right)=F_{\mathrm{RIS}}\left(c\right)+\sum^{N_{s}}\mu_{i}\left(c\right)s_{i}+\sum_{i>j}^{N_{s}}\epsilon_{ij}\left(c\right)s_{i}s_{j}+\sum_{i>j>k}^{N_{s}}\tau_{ijk}\left(c\right)s_{i}s_{j}s_{k}+\ldots$$

where $N_{s}$ is the number of protanable sites and $F_{\mathrm{RIS}}\left(c\right)$ is the free energy of the uncharged molecule, corresponding to the RIS model energy in Equation (28). The SBRIS model is thus the extension of the RIS model to include the ionization degrees of freedom. In Equation (51), we have assumed that the PE is a polybase, so that the ‘deprotonated’ corresponds to ‘uncharged’ state. However, the main ideas can be readily extended to polyacids and polyampholytes [32].

The relevant physical quantity is the probability of a particular conformational-ionization state or *rotomicrostate*

(52) $$P_{\mathrm{SBRIS}}\left(s,c\right)=\frac{e^{-\beta F_{\mathrm{SBRIS}}\left(s,c\right)}}{\Xi_{\mathrm{SBRIS}}}\;\;;\;\;\Xi_{\mathrm{SBRIS}}=\sum_{\left\{s\},\{c\right\}}e^{-\beta F_{\mathrm{SBRIS}}\left(s,c\right)}$$

where $\Xi_{\mathrm{SBRIS}}$ is the semi-grand canonical SBRIS partition function. Note that the conformational and ionization properties of the PE can be calculated from Equation (52) on equal footing.

Let us showcase the SBRIS method for the case of a linear chains with only short-range interactions, in which transfer matrix methods can be used. In this case, the SBRIS partition function can be expressed as

(53) $$\Xi_{\mathrm{SBRIS}}=\sum_{\left\{s\right\}}\Xi_{\mathrm{RIS}}\left(s\right)\;\;;\;\;\Xi_{\mathrm{RIS}}\left(s\right)=\sum_{\left\{c\right\}}e^{-\beta F_{\mathrm{SBRIS}}\left(s,c\right)}$$

where $\Xi_{\mathrm{RIS}}\left(s\right)$ is the RIS partition function of a molecule in a *frozen* protonation state *s*. Using the RIS machinery, $\Xi_{\mathrm{RIS}}\left(s\right)$ can be expressed in terms of transfer matrices

(54) $$\Xi_{\mathrm{RIS}}\left(s\right)=pU_{1}^{s_{1}s_{2}}U_{2}^{s_{2}s_{3}}\cdots U_{N}^{s_{N-1}s_{N}}q^{T}$$

The matrices $U_{i}^{s_{i}s_{i+1}}$ now depend on the ionization states of the sites pending from the ends of the *i*th bond, and they include extra free energy terms due to protonation. For instance, $U_{i}^{00}=U_{i}^{10}=U_{i}$ correspond to the addition of a bond with a neutral site so the transfer matrix $U_{i}$ is given by Equation (31). However, $U_{i}^{01}$ represents the addition of a bond with a protonated site to a bond with a neutral group. The additional protonation free energy is introduced, multiplying by the reduced activity, $U_{i}^{01}$ = $U_{i}z$. Finally, if a bond with a charged site is added to a bond with a charged site, we must use the matrix

(55) $$U_{i}^{11}={\left(\begin{array}{ccc} u_{t} & \sigma u_{g+} & \sigma u_{g-}\\ u_{t} & \sigma \omega u_{g+} & \sigma \psi u_{g-}\\ u_{t} & \sigma \psi u_{g+} & \sigma \omega u_{g-} \end{array}\right)}_{i}z$$

where $u_{\nu }$ represents the interaction between nearest-neighboring sites through a bond in state ν (i.e., *t*, *g*+ or *g*−).

The sum over the protonation states in Equation (53) can be analytically performed as [25]

(56) $$\Xi_{\mathrm{SBRIS}}=\sum_{\left\{s\right\}}\Xi_{\mathrm{RIS}}\left(s\right)\;\;=\sum_{\left\{s\right\}}U_{1}^{s_{1}s_{2}}U_{2}^{s_{2}s_{3}}\cdots U_{N}^{s_{N}s_{N+1}}=I\prod_{i}V_{i}I^{T},$$

where V and I are the super-matrix

(57) $$V=\left(\begin{array}{cc} U^{00} & U^{01}\\ U^{10} & U^{11} \end{array}\right).$$

and $I=\left(E\;E\right)$. E denotes the identity matrix (thus containing ones in the diagonal and zeros elsewhere). The addition of matrices in Equation (56) has been performed using the following trick. In Equations (33) and (48), one calculates the corresponding partition function as a sum of products of Boltzmann factors. However, in Equation (56) one needs to sum products of matrices. One can express this sum of products of matrices as a simple product of matrices by defining ’transfer matrices of matrices’ which is the matrix V. In this way, the SBRIS partition can be calculated introducing Equations (56) and(57) in Equation (53) as in (53), the SBRIS partition stays

(58) $$\Xi_{\mathrm{SBRIS}}=p^{\prime }\prod_{i}V_{i}q^{\prime }T$$

with $p^{\prime }=\left(p\;p\right)$ and $q^{\prime }=\left(q\;q\right)$. In summary, we obtain the same expression that for the RIS partition function but replacing $U_{i}\to V_{i}$, $p\to p^{\prime }$ and $q\to q^{\prime }$. With the SBRIS partition function at hand, one can compute the degree of protonation and the bond state probabilities using Equations (34) and (46).

In general, we can calculate any conformational average that can be expressed as a product of transfer matrix. As discussed in Section 2.2.2, this is the case of the radius of gyration and end-to-end distance, among others. First, we express the sought binding/conformational average as

(59) $$\langle f\left(s,c\right)\rangle =\sum_{s,c}\frac{e^{-\beta F_{\mathrm{SBRIS}}\left(s,c\right)}}{\Xi_{\mathrm{SBRIS}}}f\left(s,c\right)=\sum_{s}\frac{\Xi_{\mathrm{RIS}}\left(s\right)}{\Xi_{\mathrm{SBRIS}}}{\langle f\left(s,c\right)\rangle }_{c}$$

where one identifies

(60) $${\langle f\left(s,c\right)\rangle }_{s}=\sum_{c}\frac{e^{-\beta F_{\mathrm{SBRIS}}\left(s,c\right)}f\left(s,c\right)}{\Xi_{\mathrm{RIS}}\left(s\right)}$$

as the average of $f\left(s,c\right)$ over the conformations of the frozen ionization state *s* and the factor $\Xi_{\mathrm{RIS}}\left(s\right)/\Xi_{\mathrm{SBRIS}}$ as the probability of the ionization state *s*. Now, we can use all the machinery of the RIS model to compute ${\langle f\left(s,c\right)\rangle }_{s}$. For instance, if ${\langle f\left(s,c\right)\rangle }_{s}=R_{g}^{2}\left(s\right)$ is the radius of gyration at the fixed ionization state *s*, Equation (35) leads to

(61) $$R_{g}^{2}\left(s\right)=\frac{2}{M\Xi_{\mathrm{RIS}}\left(s\right)}J\prod_{i=1}^{N}S_{i}^{s_{i}s_{i+1}}J^{\prime }$$

where $S_{i}^{s_{i}s_{i+1}}$ are the super-matrices as defined in Equation (36), now expressed in terms of the matrices $U^{s_{i}s_{i+1}}$ introduced above. Replacing Equation (61) in Equation (59) and using the same matrix summation trick as in Equation (56), the radius of gyration stays

(62) $$R_{g}^{2}=\frac{2}{M\Xi_{\mathrm{SBRIS}}}H\prod_{i=1}^{N}\Sigma_{i}H^{\prime }$$

where $H=\left(J\;J\right)$, $H^{\prime }=\left(J^{\prime }\;J^{\prime }\right)$ and

(63) $$\Sigma_{i}={\left(\begin{array}{cc} S^{00} & S^{01}\\ S^{10} & S^{11} \end{array}\right)}_{i}$$

Note that the resulting radius of gyration, which is pH-dependent, is obtained by simply replacing $\Xi_{\mathrm{RIS}}\to \Xi_{\mathrm{SBRIS}}$, J→H and $S_{i}\to \Sigma_{i}$ in the RIS expressions Equations (35)–(39). Therefore, we have a recipe to extend the RIS formalism to ionizable systems. Proceeding in the same way, other quantities such as the end-to-end distance, average square distances between sites and optical polarization, among others [85], can be evaluated as functions of the pH-value.

**Figure 4:**
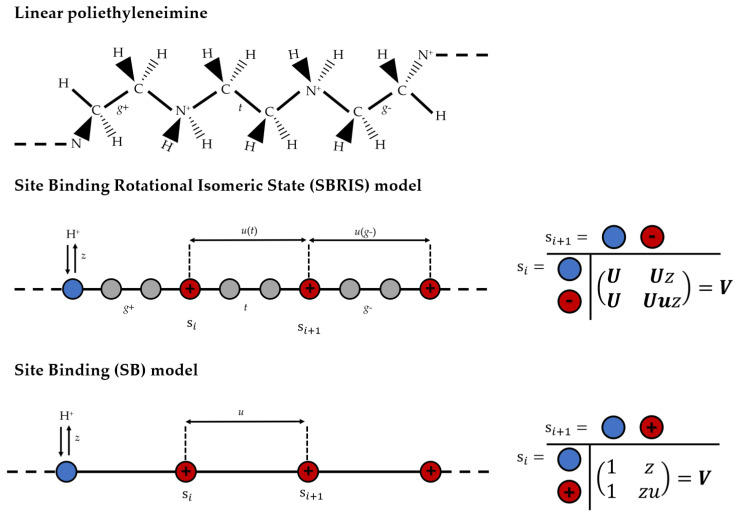
**Upper** panel: Atomistic representation of linear polyethylenimine (LPEI). Within the RIS framework, three different bonds are identified: nitrogen–carbon, carbon–carbon, and carbon–nitrogen. Correspondingly, three transfer matrices must be ascribed. **Middle** panel: SBRIS model for LPEI. Only three rotational states are considered, the ones with minimum energy (trans, gauge+ and gauge−) which are included in the RIS transfer matrix *U*. **Lower** panel: SB model of LPEI. The molecule is considered as a set of protonating sites without conformational structure. Color code in all panels: Unprotonated site (blue), protonated site (red), inert site (grey). Figure reprinted with permission from the PhD. thesis of Pablo M. Blanco [97].

Let us illustrate the theoretical models introduced in this section using linear poly-ethyleneimine (LPEI) as a model of weak PE. By analyzing the chemical structure of LPEI, which is depicted in the upper panel of Figure 4, one identifies three kinds of bonds. From left to right, these bonds join nitrogen–carbon, carbon–carbon and carbon–nitrogen atoms, respectively. Therefore, within RIS formalism, three Flory U-matrices are necessary. The ionization degrees of freedom can be included by using the SBRIS, which permits to study ionization and conformational degrees of freedom on equal footing as outlined in Figure 4 (middle panel). Then, the Boltzmann factors corresponding to the protonation energy *z* and to the pair interactions between protonated groups (u(t) and u(g)) are included in the Flory U-matrices forming the SBRIS V-matrices. Note that the electrostatic interactions differ for each conformational state. As a result, $u_{t}$ and $u_{g}$ have different values.

**Figure 5:**
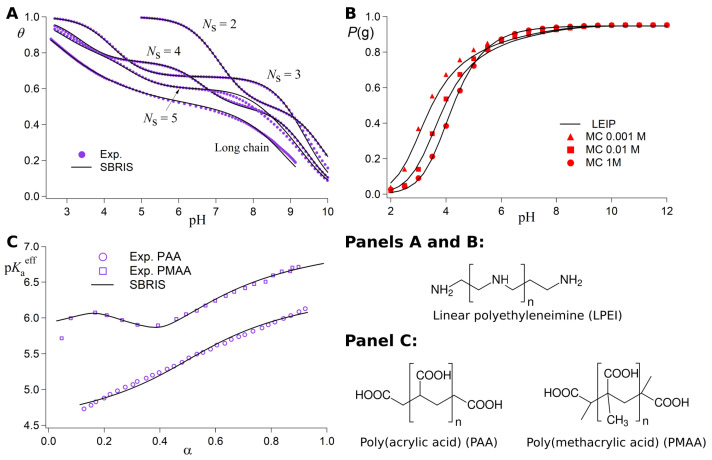
Panel (**A**): Degree of protonation θ as function of pH of linear polyethyleneimine obtained by potentiometric titration (circles) corresponding to the oligomers with $N_{s}=\;$2, 3, 4, 5 amines and to a long chain. The corresponding best fit (lines) of each curve to the Site Binding Rotational Isomeric State model follows closely the experimental data. Panel (**B**): Probability of finding a carbon–carbon bond of LPEI in a *gauche* state P(g) as a function of pH at various ionic strength values for a model LPEI. The model was independently solved by (i) a combination of SBRIS and LEIP formalisms (lines) and Monte Carlo (MC) simulation (markers). Panel (**C**): Effective p$K_{a}$ of PAA (circles) and PMAA (squares) measured by potentiometric titration together with their corresponding best fit to the SBRIS model (lines) as a function of the degree of ionization α (note that in this case, α=1−θ). Each panel is reproduced with permission from the corresponding sources: Panel (**A**) from Ref. [25] (Copyright (2014) from the Royal Society of Chemistry), panel (**B**) from Ref. [68] (Copyright (2019) from the Multidisciplinary Digital Publishing Institute) and panel (**C**) from Ref. [32] (Copyright (2014) from the American Chemical Society).

In Figure 5A, the experimental titration curves for LPEI of different lengths *N* (purple markers) are shown together with the ones corresponding to the best-fitting SBRIS model (continuous lines) [25]. Remarkably, all the curves obtained only included short-range interactions. However, the SBRIS formalism can be coupled to the LEIP method described in Section 3.1.2 in order to also include long-range interactions. The procedure is very similar to what is illustrated in that section but correcting not only the chemical potentials but also the conformational energies. The resulting formalism was recently validated against constant pH Monte Carlo (cpH) simulations including electrostatics and excluded volume effects [140]. The LEIP-corrected SBRIS (black lines) matched the results from the cpH simulation (red markers) for ionic strengths ranging from 0.001 M to 1 M, as can be observed in Figure 5B for the probability to find a C–C bond in a *gauche* state. Combining the LEIP method with the SBRIS formalism opens the possibility to design pH-dependent new force fields to deal with both short- and long-range interactions on equal footing.

The SBRIS formalism is specially well suited to study PEs whose ionization and conformational properties are highly coupled. Let us consider the case of poly(methacrylic) acid (PMAA) and poly(acrylic) acid (PAA). These two PEs differ in the presence of an extra hydrophobic pendant methyl group in the monomeric unit of PMAA. Due to this chemical difference, PMAA experiences a sharp conformational transition in a narrow range of pH-values which is absent in the case of PAA. The conformational change results in a non-monotonic behavior of the experimental effective p$K_{a}$-value, shown in Figure 5B, not observed in the case of PAA. A proper SBRIS model (lines) was able to fit the experimental measurements of both PEs. Up to our knowledge, this is the only attempt to build a quantitative and chemically detailed model (including not only p$K_{a}$-values but also pair interactions and conformational energies) from the PMAA experimental titration curves.

Finally, note that the SB model offers a less detailed level of description than the SBRIS model as outlined in Figure 4 (bottom panel). This means that the SB cluster parameters (*z*, *u*, … in Equation (44)) should be obtained from the SBRIS parameters by means of proper averages over the conformational states. The resulting relations or “contraction equations” were derived in Ref. [32]. They present a non-trivial form and some non-evident phenomena arise. For instance, one can find triplet interactions at the SB level which are not present at the SBRIS description [25,32]. This phenomenon has been experimentally observed in the analysis of LPEI titration curves [137,142].

## 4. Computer Simulations of Weak Polyelectrolytes at Constant pH

The theoretical treatments discussed in the previous section are very useful in the understanding of the non-trivial interplay between protonation reactions and conformational equilibria. However, the complexity of PE systems makes in many cases exact analytical solutions unavailable, so that computer simulations have become a popular tool among researchers. As a result, a broad offer of software is currently available. The main challenge of computational techniques is to sample a large configurational space in the presence of competing phenomena, such as electrostatic interactions, thermal fluctuations and reversible reactions. As a consequence, simulations of weak PEs usually are rather time-consuming.

All-atom hybrid Quantum Mechanics/Molecular Mechanics (QM/MM) offer a very detailed description of the system, although they are currently restricted to very few reactive groups due to their high computational cost [143]. Alternatively, hybrid Molecular Dynamics/Monte Carlo (MD/MC) methods have been proposed in which the MD trajectory is periodically interrupted to perform MC attempts to switch a protonation state [39,144,145]. Although hybrid MD/MC simulations permit to sample a bigger number of reactive groups than in QM/MM techniques, their computational cost is too high to sample systems in which the conformational and ionization degrees of freedom are highly coupled [146]. Biased sampling techniques such as λ-dynamics can be applied to study bigger systems, but they require to define a reaction coordinate. In the case of PE systems, such a reaction coordinate corresponds to the protonation of an specific group at a given pH. λ-dynamics is available in the popular software GROMACS and CHARMM [147,148]. Weak PEs are molecules with typically hundreds of reactive groups whose ionization needs to be sampled simultaneously with the configurational dynamics of the macromolecule, which is usually too demanding for these Molecular Dynamics techniques.

Alternatively to all-atom techniques, coarse-grained models reduce the resolution of the system by encapsulating several atoms into bigger particles with an effective volume. The solvent is often treated implicitly and replaced by a proper dielectric constant in the Coulomb potential (Equation (15)). As a result, they are much less time-consuming. Monte Carlo (MC) techniques are especially well-suited to simulate coarse-grained systems since they allow a much faster sample of the configurational space. The price to pay is that often only equilibrium properties are obtained, while most information about the dynamics is lost. In exchange, MC simulations are able to reach larger time and length scales, allowing the simulation of macromolecular systems with hundreds of reactive groups. The Reaction Ensemble [149,150] and the constant-pH ensemble [139] are examples of these MC techniques.

In weak PE systems, the pH-value usually is the most relevant control variable so that it is often convenient to fix it throughout the simulation. Examples of MC techniques with this property are the constant-pH Ensemble (cpH), the Reaction ensemble [139] and the Fast Proton Titration Scheme (FTPS) [151,152]. Examples of software that allow to use some or all of these techniques are ESPResSo [153], AMBER [154,155], MOLSIM [156], FAUNUS [157,158], and LAMMPS [159]. Due to their popularity, we briefly discuss the main trends of cpH techniques and we refer the reader to the recent review by Landgesell et al. [21] for a more complete discussion of their properties, applications and differences from the Reaction Ensemble approach.

The constant pH ensemble (cpH) was designed by Reed and Reed to study acid–base equilibria in weak PEs [139]. Since in the cpH, one considers a system in equilibrium with a reservoir at a fixed proton chemical potential $\mu_{H}$, but otherwise with all other chemical potentials free, cpH simulations have also been regarded as Semi-Grand Canonical Monte Carlo simulation [24,29,56,57,68,140,160,161,162]. The inputs of cpH simulations are the pH-value and the p$K_{a}$-values of the ionizable groups. The outputs are the average number of protonated and unprotonated groups at the given pH-value, from which the relevant ionization properties introduced in Section 2.1.1 can be calculated.

Originally, the cpH method was designed to simulate a weak PE using an implicit description of the solvent and the background ions so that the DH potential (Equation (17)) was used. The probability of accepting a trial MC titration move reads

(64) $$P_{\mathrm{cpH}}=\min\left[1,\exp\left(-\frac{\Delta U}{k_{B}T}+\xi \ln\left(10\right)\left(\mathrm{pH}-pK_{a}\right)\right)\right],$$

where ΔU is the variation of the potential energy, usually dominated by electrostatic interactions, and ξ is the extent of the protonation such as ξ=+1 if a group is protonated and ξ=−1 otherwise. Note that the pH-value as input in Equation (64) is decoupled from the value of the ionic strength *I* used as input in Equations (17) and (18). This point can be problematic at extreme pH-values, since the concentration of $H^{+}$ or ${\mathrm{OH}}^{-}$ ions is high enough to surpass the concentration of background ions, potentially leading to inconsistencies between the value of *I* and the pH-value. Therefore, one should be careful to adapt the value of *I* in order to account for protons and hydroxide ions implicit in the chosen pH-value.

cpH simulations were later extended to include explicit ions in the simulation box. To preserve the electroneutrality of the system, the titration moves are coupled with the explicit addition or deletion of a “neutralizer” ion. In order to account for periodic boundary conditions, the scheme is coupled to methods such as the $P^{3}M$ [163]. The main advantage of this approach is to avoid the approximations inherent to the DH potential, which arises from a mean field-theory which breaks down at high concentrations and in presence of multivalent ions [3]. However, one needs to be aware of some additional artifacts that come as a consequence of this implementation. Again, the concentration of the explicit neutralizer ions is decoupled from the pH-value, which can lead to the same artifacts at extreme pH-values previously commented. Furthermore, this “neutralizer” ion should not be confused with explicit $H^{+}$ or ${\mathrm{OH}}^{-}$ ions since the concentration of neutralizer ions does not necessarily correspond with the one corresponding to the pH-value. In addition, one needs to correct Equation (64) by the excess chemical potential of the neutralizer ion [164,165]. Otherwise, one obtains deviations that are asymmetric depending whether a cation or an anion is used as neutralizer ion. Such deviations are small when there are only monovalent ions in the system but they can become significant in the presence of multivalent ions.

The above-mentioned techniques are conceived for single-phase systems, such as solutions of macromolecules. However, in two-phase systems where one or more components cannot be exchanged between them, the Donnan partitioning of ions needs to be considered together with charge regulation. An important consequence of Donnan partitioning is that it also affects the concentration of protons so that the pH-value is not necessarily equal in the two phases. Only recently, computational methods have been developed allowing to simulate such systems [38,166]. In particular, the Grand Reaction method [38] has revealed promising applications in the simulation of weak hydrogels [167,168,169,170] and potentially could be used to simulate other two-phase systems such as coacervates and dialysis solutions of proteins.

## 5. The Role of Charge Regulation in the Elasticity of Weak Polyelectrolytes

The conformational models introduced in Section 2.2.3 no longer apply to charged macromolecules due to the important role of electrostatic in the chain structure and the long-range nature of coulombic interactions [171,172]. For instance, the stretching behavior of single-stranded nucleic acids, often used as paradigmatic models of strong PEs, strongly depends on the concentration and valency of the salt ions in solution [101,116,173,174,175,176,177,178]. Salt ions screen the repulsive intra-molecular electrostatic interactions within the PE chain, causing it to be more easily stretched in conditions of low ionic strength. Due to electrostatic interactions, new elastic regimes arise. Monte Carlo simulations suggest that two different length scales are relevant to explain the elastic behavior of strong PE, a feature that is well captured by the so-called “Snake-like model”, a name first proposed by Ullner [31,78,179]. At low forces and at a long length scale, strong PEs behave as a set of swollen electrostatic blobs while at large forces a short-ranged wrinkled structure, stabilized by condensed ions, is detected [180,181,182]. A very complete review of the existing theories can be found in Ref. [116]. In all these studies, the charge is a static parameter independent of the applied force.

However, in the case of flexible weak PEs, the application of an external force can modify the distance between charged groups and thus the electrostatic interactions. As a result, CR can in principle take place. However, the AFM experiments available in the literature concerning weak PEs either focus on other variables such as the temperature [104] or were carried out at pH conditions where CR is negligible [183]. Preliminary AFM experiments done in our research group suggest a moderate effect of pH on the force-extension curve of hyaluronic acid [184,185]. More evidence is thus needed to fully understand the issue, and more experimental work on this topic would be desirable. Only a few recent computational studies address this fundamental question [29,68], which we briefly summarize here.

**Figure 6:**
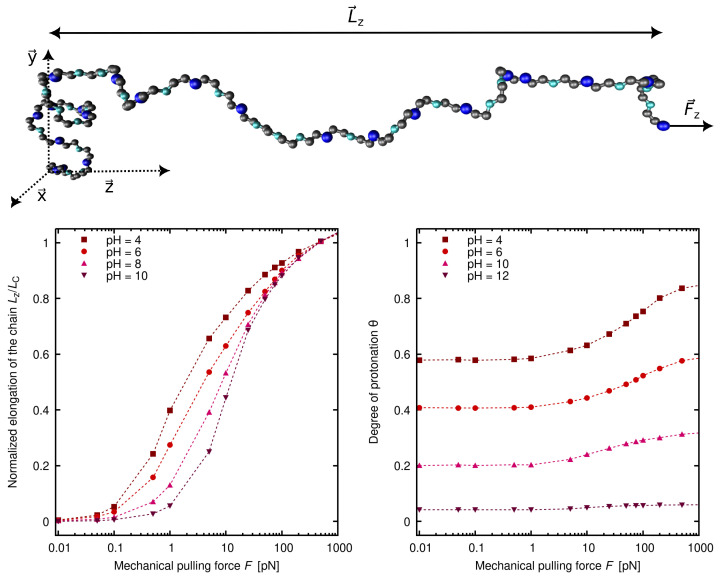
**Upper** panel: Snapshot from a constant pH Monte Carlo simulation of the stretching of linear a polyethylenimine chain. The force is applied in the *z*-direction. Color code: aliphatic group (grey), protonated amino group (blue), deprotonated amino group (cyan). **Lower** panels: Normalized elongation of the PE chain $L_{z}/L_{C}$ (left) and degree of protonation θ (right) as functions of the force *F* at different pH-values. Markers denote simulation data while the continuous lines are added to guide the eye. Figure adapted with permission from Ref. [29] Copyright 2019 American Chemical Society.

cpH simulations [29,68] suggest a significant influence of the pH-value on the force-extension curve. This effect is larger at pH-values for which the CR capacity is maximum, in accordance to the arguments exposed in Section 2.1.1. A simplified SBRIS model of linear poly(ethylenimine) (LPEI) was used, with three possible rotational states for the bonds (*t*, *g*+ and *g*−). Excluded volume, electrostatic interactions and charge regulation effects were included. A snapshot of the simulations is shown in Figure 6. In the example, hydrogen bonding between two consecutive uncharged amine groups is considered when the carbon–carbon bond is in the gauge state. The chain extension as a function of the applied force is shown in the left lower panel of the same figure, for pH-values ranging from four to ten. The effect of the pH-values is significant at intermediate forces ranging from ∼1 pN to ∼100 pN. For these force values, the mechanical work is of the same order of magnitude of the rotational free energy barrier from the gauge to the trans state. An important consequence of this fact is that the persistent length is no longer a constant throughout the stretching process, as observed in stiffer PE such as DNA, but force-dependent [29].

The degree of protonation θ becomes a force-dependent quantity, as observed in the lower right panel of the same figure. θ increases as the PE is stretched due to the decrease in the electrostatic repulsions. At pH=4, for which the charge regulation capacity is maximum, the observed increase is around 20%. Therefore, the effect of an external force can induce the binding and release of protons. Another interesting feature of the stretching of weak PEs is that their scaling behavior at low forces differs from the one expected both from a strong PE or a neutral polymer (cf. Equation (43)). The Pincus scaling exponent ν obtained from the cpH simulations was found to be dependent on the pH and ionic strength [29]. For low-enough pH-values, the macromolecule is fully charged and ν=3/5, as expected from a strong PE. In increasing pH-value, however, ν decreases until the ν equates 1/2, the expected value for a neutral chain. This transition is observed even in the absence of charge fluctuations [68] and seems to be determined by the charge density.

The coupling of binding and stretching has been also discussed in the context of ligand-receptor biochemical systems [186,187,188]. The analytical model proposed by Radiom and Borkovec consisting of a FJC with interacting sites predicts force-induced bound-unbound ligand transitions [121]. The coupling between binding and mechanical stretching opens exciting possibilities for the design of smart devices able to capture or release molecules into the media by stretching or compressing PE chains.

## 6. Adsorption of Weak Polyelectrolytes onto Charged Objects

Electrostatic interactions are the leading force in the adsorption of polyelectrolytes (PE) onto charged objects such as nanoparticles, colloidal particles, proteins and other macromolecules. The expected picture is that macromolecule and surface present a sufficient net charge of opposite sign, so that the PE is strongly adsorbed. However, many other factors influence adsorption which lead to counter-intuitive behaviors, such as adsorption onto an object with the same net charge as the PE. For the sake of clarity, we discuss the adsorption of single and multiple chains in different sub-sections.

### 6.1. Adsorption of a Single Chain

With a single weak PE chain interacting with a charged object, two competing effects determine the adsorption of the PE. First, electrostatic attraction between oppositely charged groups in the PE and in the charged object leads to the PE adsorption onto the charged object. The PE adsorption into the charged object in turn triggers the ionization of the PE, increasing its net charge. Second, the intra-molecular electrostatic repulsion between like-wise charged groups in the PE chain limits the ionization of the PE. This hinders the PE adsorption onto the charged object at a given pH sometimes requiring extreme pH-values for the PE to acquire a sufficient charge to adsorb into the charged object [189]. In short, the adsorption of the weak PE can be promoted by tuning the conditions under which the PE-charged object attraction is favored and simultaneously the intra-molecular electrostatic repulsion within the PE chain is minimized. The variables to be adjusted are the pH-value and the ionic strength of the solution and the surface charge density of the charged object.

Charge regulation is thus a key feature on the adsorption of PE onto charged object, which can be present in only one component (the PE or the charged object) or in both of them. Many authors have investigated such systems, usually considering either a weak PE or a weak charged object [190,191,192,193,194,195,196,197]. Studies on the adsorption of weak PE adsorbing onto a strong charged object have focused on the influence of parameters such as the properties of the PE chain (stiffness, length) [193,198,199] and properties of the added salt (concentration and valency) [193,198,200,201,202]. Studies can be found also of the complementary case, a strong PE adsorbing onto a weak charged object, including adsorption of the strong PE onto Janus particles [203], nanoparticles [162,204,205] and globular proteins [70,74,206,207,208]. The case when both the PE and the charged object can charge-regulate has only recently been studied using constant pH simulations [209,210,211]. They mutually enhance each other’s ionization, ultimately leading to different features than when only one component can charge-regulate. For example, different adsorption patterns were found in PE-charged object complexes depending on whether the PE, the charged object, or both were strong or weak PE [210]. The difference in the p$K_{a}$-values of the acid and base groups of the PE and charged object is a key parameter determining how important are charge regulation effects [58,210,212].

Non-intuitive physics can arise when either the PE or the charged object have multiple types of acidic and basic groups. For example, PEs can adsorb onto surface even when both have the *same* sign in the net charge. Since such attractive interaction is not intuitively expected under these conditions, it is often described as complexation/adsorption in the ’wrong side’ of the isoelectric point (WSIP) [213]. Adsorption in the WSIP has been mainly observed in proteins, which can be considered a subclass of weak polyelectrolytes that contain different types of titratable groups (acidic and basic groups with varying p$K_{a}$ values) within their three-dimensional structure. There are two mechanisms that can explain adsorption of proteins in the WSIP of a protein. The first mechanism is charge regulation. The interaction with the charged object triggers a change of the sign of the protein charge by shifting the protonation equilibria. As a result, near the surface, the protein and the charged object present opposite charge signs. The second mechanism is the heterogenous charge distribution along the protein chain or patchiness. For instance, a PE can adsorb onto a patch with an opposite charge sign to the one of the net protein charge. Adsorption of PE in the WSIP of proteins has been reported due to charge regulation [37,70,209,214], to charge patchiness [206,213,215,216,217,218,219,220] or to both mechanisms acting cooperatively [71,221,222]. A general framework has been recently proposed which uses the charge regulation capacity and the dipolar moment as key parameters to predict under which conditions should prevail the charge regulation or charge patchiness mechanism [71].

An illustrative example of both mechanisms producing the adsorption of a PE onto a protein in the WSIP can be found in the study by Torres et al. [208] They considered the case of β-lactoglobulin, a protein found in cow milk, interacting with strong cationic and anionic polyelectrolytes. Using a coarse-grained model and constant pH simulations, the adsorption of the PEs onto the protein at different pH-values was analyzed. They observed a shift in the isoelectric point of the protein due to charge regulation in presence of the PE chain, especially in the case of a cationic PE. Furthermore, it was detected that the PE chain was adsorbed in different regions of the protein depending whether the PE was cationic or anionic, highlighting the importance of the charge patchiness mechanism. Both observations evidence that both mechanisms can act together to allow the adsorption of the PE at the WSIP of the protein.

**Figure 7:**
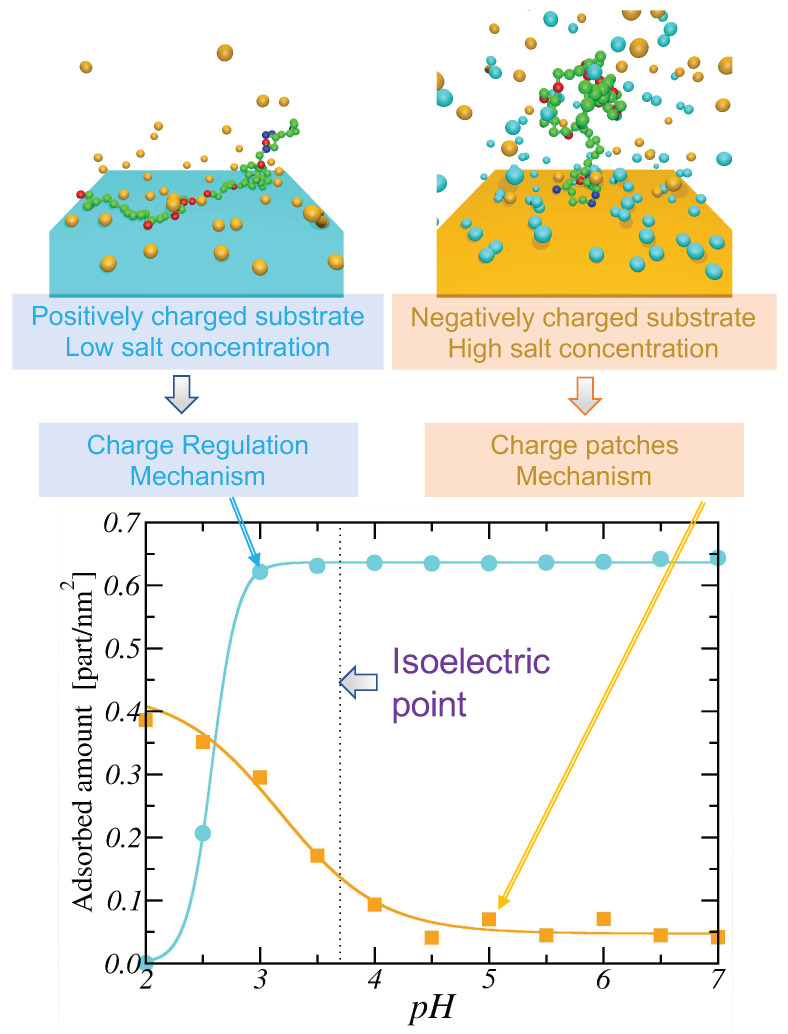
**Upper** panels: Snapshots from constant pH simulations of a coarse-grained model of casein macropeptide (CMP) in a solution with explicit ions in the presence of a surface with a positive (right) and negative (left) charge density. Color code: neutral aminoacid (green), acid aminoacid (red), basic aminoacid (dark blue), positively charged surface (cyan), negatively charged surface (orange), small anion (ochre) and small cation (light blue). **Lower** panel: Adsorbed amount of CMP as a function of the pH-value. The isoelectric point of the CMP in bulk solution (${\left(pH\right)}_{\mathrm{iso}}\approx 3.7$) is depicted as a vertical line. Note that for a positively charged surface, significant adsorption is observed for ${\left(pH)<(pH\right)}_{\mathrm{iso}}$, where CMP is also positively charged. The responsible mechanism of this fact is charge regulation. On the other hand, for a negatively charged surface (orange), adsorption is present at ${\left(pH)>(pH\right)}_{\mathrm{iso}}$, for which the chain is also negatively charged. In this case, the mechanism is charge patchiness. Reprinted from [19], Copyright (2022), with permission from Elsevier.

Recently, adsorption in the WSIP has also been reported for a flexible peptide, the casein macropeptide (CMP) [19]. Conversely to globular proteins, which typically have stable tridimensional structures, CMP has a very flexible structure and it is classified as an intrinsically disordered protein (IDP) [223,224]. Another interesting feature of CMP is its patchy charge distribution, consisting of a cationic head and a long anionic tail. In a recent work, a coarse-grained model and constant pH simulations was used to study the adsorption of CMP onto a charged substrate, which was modelled as a charged plane. Two snapshots of the simulations are shown in the upper panels of Figure 7 for the case of a positively (left panel) and a negatively (right panel) charged surface. As can be observed in Figure 7 (lower panel), CMP adsorbs in the WSIP in both cases. However, in each case the mechanism responsible for such adsorption is different, thereby leading to different conditions in which the adsorption on the WSIP is favored. In the case of the positively charged substrate, CMP adsorbs on the WSIP due to a strong charge regulation effect on its acidic groups which shifts the isoelectric point of CMP. Consequently, adsorption on the WSIP is favored at low salt concentration where the plane-CMP interaction is less screened by the salt. In the case of a negatively charged surface, the preponderant mechanism is charge patchiness. CMP adsorbs on the WSIP through its cationic head adopting a tail-like conformation. In this case, adsorption on the WSIP is favored at high salt concentrations for which the repulsion between the plane and the like-wise charged patch on the CMP chain is screened. To sum up, the interplay between charge regulation and charge patchiness is not trivial and can lead to different behaviors even for the same molecule at similar pH-values.

### 6.2. Adsorption of Multiple Chains

The interaction of multiple polyelectrolyte (PEs) chains with charged colloidal objects usually leads to highly complex phenomena such as aggregation, self-assembly, macromolecular complexation, coacervation, gelation, precipitation and in general, to a phase separation between a polymer-rich phase and a polymer-poor phase. Experimentally, methods such as atomic force microscopy, optical tweezers and scattering techniques are typically used to characterize the size and structure of such systems [196]. Theoretically, the interaction between multiple colloidal particles has been described using the classical Derjaguin, Landau, Verwey and Overbeek (DLVO) theory which calculates the force between the particles in suspension under the mean field approximation. More modern theoretical methods such as self-consistent field theory, variational methods, density functional theory or numerical modelling include ion–ion correlations and the effect of PEs in solution in the interaction between colloidal particles [192,225,226,227,228].

Constant pH simulations have provided valuable insights into the role of charge regulation on the adsorption of multiple weak PEs onto charged objects. For example, the solution conditions (pH and ionic strength) have a dramatic effect on the structural properties of the PE-nanoparticle complexes sometimes leading to overcharging [193]. For systems of weak PEs bridging nanoparticles, variations in the polyacid chain length and concentration, as well as the polyacid-to-nanoparticle mixing ratio, were found to influence the ionization properties [229]. Electrostatically stabilized complexes between weak polyacids and macroions have been reported for pH-values above the p$K_{a}$-value of the polyacid [230].

**Figure 8:**
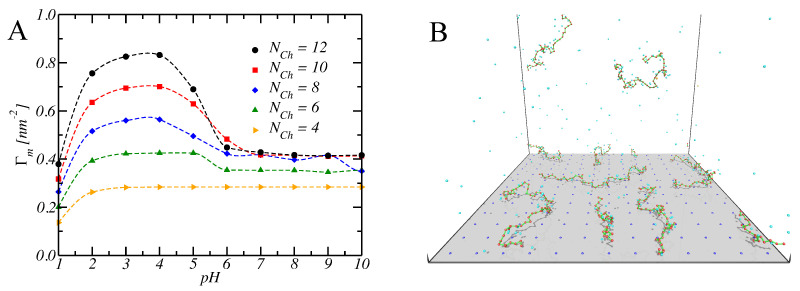
Panel (**A**): Fraction of the charged surface covered by polyelectrolyte chain $\Gamma_{m}$ as a function of pH-value for systems containing a different number of PE chains $N_{\mathrm{Ch}}$ obtained by means of constant pH Monte Carlo simulations. Panel (**B**): Snapshot from a simulation with $N_{\mathrm{Ch}}=12$, pH=7 and salt concentration 1 mM. Reprinted from Ref. [232] Copyright (2021), with permission from Elsevier.

cpH simulations allow to rationalize non-intuitive experimental observations. For example, the adsorbed amount of polyacrylic acid (PAA) onto positively charged polystyrene latex nanoparticles was found to follow a non-monotonic behavior when varying the pH of the solution [231]. In particular, a maximum in the adsorbed amount was measured at intermediate pH-values below the the p$K_{a}$-value of PAA. Recently, constant pH simulations were used to investigate this system using a coarse-grained model including flexible weak PE chains and a charged plane as substrate [232]. All the titratable monomers of the PE were considered to be identical with the same p$K_{a}=4.25$ and only electrostatic and excluded volume interactions were included. For a sufficient number of PE chains $N_{\mathrm{Ch}}$, the computer simulations qualitatively reproduced the non-monotonic behavior as can be observed in Figure 8 (Panel A). The fundamental mechanism is that, when the PE chains are sufficiently charged, the lateral repulsion between PE chains lead to the desorption of some PE chains. This results in a higher adsorbed amount of PE chains at intermediate pH-values where the PEs are charged enough to be attracted by the substrate but not sufficiently charged to repel each other on the surface. This situation in which some PEs are adsorbed onto the substrate while others are free in solution corresponds to the simulation snapshot at pH=7 shown in Figure 8 (Panel B). Ultimately, the adsorption of PEs into the surface leads to an overcharging of the substrate due to exchange of adsorbed anions by the PE chains.

## 7. Weak Polyelectrolytes in Crowded Systems

Biopolymers in the cell are far from being in the dilute solution conditions under which most experiments are performed. The cell cytosol is characterized by a high concentration of macromolecules, which can occupy up to 20–40% of the volume. In biotechnological applications, solutions with a high concentration of protein are often used. For instance, excipient solutions with a protein concentration exceeding 100 g/L are often used during ultrafiltration/diafiltration processes in biopharmaceutical manufacturing [233,234,235,236]. The term “macromolecular crowding” (MCr) has been coined to describe such conditions of high concentration of macromolecules and nanoparticles [237,238,239,240]. MCr can modify the dynamical and conformational properties of macromolecules. Since conformational and ionization properties of peptides and other biopolymers can be coupled in general [241,242], interplay between crowding and charge regulation can be in principle expected.

### 7.1. Effect of Macromolecular Crowding in the Properties of Weak Polyelectrolytes

Macromolecular crowding usually acts through non-specific interactions such as excluded volume, electrostatics and hydrodynamic interactions. For instance, Fluorescence Correlation Spectroscopy (FCS) and Fluorescence Recovery After Photobleaching (FRAP) experiments both in vivo [243] and in vitro [244], track the diffusion coefficient of tracer proteins in crowded media, suggesting a reduction in the diffusion coefficient of the tracer protein by up to two orders of magnitude due to the presence of MCr. These observations have been confirmed by computer simulations [245,246,247,248,249,250,251], which also predict anomalous diffusion regimes [252,253,254,255,256]. The shape and softness of the involved particles can also play a role in diffusion under MCr conditions [257,258,259].

The effect of MCr on the conformational properties of proteins is not trivial. Although one would naively expect MCr to induce the folding of the macromolecule due to steric hindrance, actually all possible outcomes have been reported. In the case of globular proteins, macromolecular crowding induced protein unfolding in some cases and protein folding in other cases, while some proteins remained unaffected [260]. A similar myriad of cases has been reported for intrinsically disordered proteins (IDPs), which have been classified as foldable (fold upon crowding), unfoldable (extended upon crowding), and non-foldable (mostly unaffected) [261,262,263,264].

Finally, because of its impact in the diffusion and conformational properties of the biomacromolecules, MCr can affect the kinetic properties of enzymes [239,240,248,265,266,267,268,269,270]. In their pioneering work, Ogston and Laurent predicted an increase in the chemical potential of the protein when a crowding agent was added to the solution [271,272]. Minton modified the transition state theory [273] by using an apparent equilibrium constant that incorporated the activity coefficients of all reactants. As a result, a Michaelis–Menten kinetic equation dependent on the excluded volume fraction is obtained [237,274,275]. It has also been reported that both the catalytic constant and the maximum velocity are affected by MCr [268,269,276].

### 7.2. Charge Regulation Triggered by Macromolecular Crowding

Since macromolecular crowding can affect the reactivity and structure of biomacromolecules, one could expect a possible charge regulation response triggered by a sufficiently high macromolecular concentration. Only recently, this hypothesis has been examined by theoretical [73] and experimental [277,278] studies which have studied the ionization of weak polyelectrolytes and peptides under MCr, respectively. Let us first review the theoretical insights on the charge regulation response triggered by MCr and later contrast those insights with the experimental observations.

**Figure 9:**
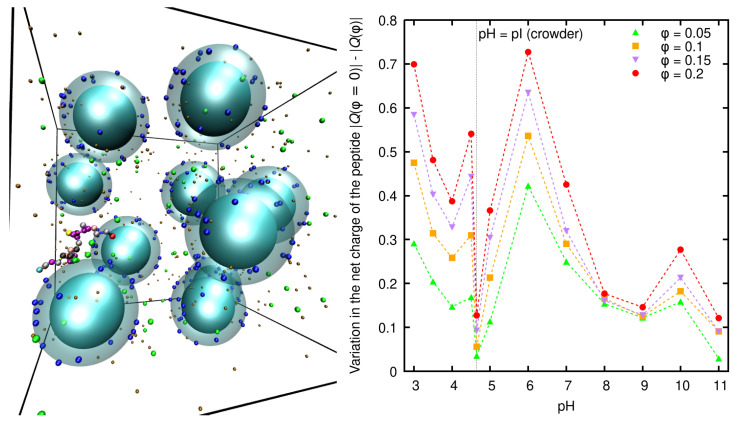
**Left** panel: Snapshot from a constant pH simulation of a coarse-grained model of histatin-5 (polymer chain) surrounded by charged crowders (colloidal particles with small charged beads on its surface). **Right** panel: Variation in the net charge of histatin-5 as a function the fraction of volume occupied by the crowders ϕ, estimated as ΔQ=|Q(ϕ=0)|−|Q(ϕ)|, as a function of pH. The added salt concentration is 0.01 M. Reprinted with permission from Ref. [73], Copyright (2021) from the Royal Society of Chemistry.

In a recent work [73], we analyzed the impact of macromolecular crowding on the ionization of two intrinsically disordered proteins (IDPs) by means of constant pH computer simulations with explicit salt ions. Histatin-5 (a polycationic-like IDP) and β-amyloid 42 (a polyampholyte-like IDP) were taken as examples of two different representative types of IDPs. In both cases, Bovine Serum Albumin (BSA) was used as crowding agent. The IDPs were considered within a coarse-grained model of bead-and-spring chains with titrable sites. BSA was treated as soft spheres under the Chain Entanglement Softened Potential (CESP) approximation [257], depicted in the simulation snapshot in Figure 9. Within this model, the crowders are treated as spheres with a hard core surrounded by a soft penetrable shell. The model included only steric and electrostatic interactions.

Upon addition of neutral crowders, we observed a variation in the charge of histatin-5 up to ∼0.6e for a volume fraction of crowders ϕ=0.5. Our results suggest that this increase is due to an effective increase on ionic strength produced since the same number of small ions are confined in a reduced available volume. In the case of charged BSA crowders, a stronger response is observed at lower ϕ-values in comparison with the situation of neutral crowders. The effect of crowding in the ionization of the IDP can be estimated by calculating ΔQ=|Q(ϕ=0)|−|Q(ϕ)|, the difference of the macromolecular charge with and without excluded volume. In Figure 9 (right panel), ΔQ is shown as a function of the pH-value for ϕ = 0.05 to 0.2. The peaks in ΔQ coincide with the pH-values of maximum charge regulation capacity, a trend that is consistently observed for the rest of the studied cases. In our study, we considered the case of short peptides with a relatively low charge density. In the case of homogenous weak PEs, which typically have a higher charge densities, we expect a more intense CR effect of MCr.

The recent potentiometric titration experiments performed by Yekymov et al. [277,278] of poly(acrylic acid) (PAA) in presence of MCr suggest significant influence of MCr on the ionization of the PE. They studied the ionization of PAA in solution with two different crowder agents: short chains of poly(vinyl alcohol) (PVA) and Carbon Black nanoparticles with PVA grafted on their surface (CB-PVA). In the first case, they observed a *negative* shift in the effective p$K_{a}$ of PAA of roughly 0.9 units due to the presence of a 13% weight fraction of PVA. This means that, for the same pH-value, PAA was found to be more ionized in presence of MCr than in its absence. This observation qualitatively agrees with the trends yielded by our cpH simulations on the ionization of histatin-5 in presence of neutral crowders. However, the authors reported a *positive* shift in the effective p$K_{a}$ of PAA of roughly 0.4 units upon addition of a 1% weight fraction of CB-PVA crowders. This results goes in the opposite direction than in the case of short chains of PVA addition since PAA is less ionized in presence of MCr than in its absence. The authors hypothesized that this fact could be due to a conformational change in PAA triggered by MCr. Those different experimental trends deserve a more detailed theoretical analysis to disentangle the different possible outcomes that CR triggered by MCr can produce on weak PEs.

The observations from these early studies suggest that MCr can induce a CR response in weak PEs and biomacromolecules. The trends gathered so far are not trivial and further experimental and theoretical efforts are needed to fully understand these phenomena. We suspect that, although so far the observed effect of MCr in CR is moderate, it could have biological implications in systems such as membrane-less organelles. In addition, the influence of MCr could probably be more intense in macromolecules with a larger charge density or in presence of multivalent ions. We consider that these studies open exciting lines for new research.

## 8. Outlooks

Although in recent years an increasing number of studies have shed light on different aspects of charge regulation, there are still many unanswered questions and challenges to be addressed.

For example, current research studies are focused on understanding CR in two-phase systems with different concentrations of weak PEs. Examples of such systems are weak polyelecrolyte hydrogels [169,170], coacervates of weak polyelectrolytes [279,280,281,282,283] and concentrated solutions of proteins under ultrafiltration–diafiltration processes [236]. In such systems, CR is coupled with the Donnan potential generated between the two phases producing a complicated feedback loop [38,165]. Recent advances in simulation techniques permitting to model those systems, such as the Grand-Reaction ensemble method, open exciting new possibilities [38,166].

Further research is also needed to understand the impact of competitive reactions such as metal binding or reversible crosslinking reactions on the ionization of weak PEs. Such research could provide new insights important for applications such as quelation of metals by PEs in wastewater [14,15,16,17]. It could be also fundamental in the understanding of the ionization of weak PEs with groups capable to create strong intramolecular cross-links, which is the case of polyboronates [284,285]. With this aim, new theoretical and simulation methods need to be developed. Some of the tools here presented, such as the Site Binding Rotational Isomeric State model [25] or the Local Effective Interaction Parameter method [79] could probably be extended to tackle such important topics.

## Data Availability

The data that support the findings of this study are available on request from the corresponding author.

## Abbreviations

The following abbreviations are used in this manuscript:

AFM	Atomic Force Microscopy
CMP	Casein MacroPeptide
cpH	constant pH Monte Carlo (cpH)
CR	Charge Regulation
DH	Debye–Hückel
DLVO	Derjaguin–Landau–Verwey–Overbeek
DNA	DeoxyriboNucleic Acid
FJC	Freely Jointed Chain
FRC	Freely Rotating Chain
FTPS	Fast Proton Titration Scheme
FWPE	Flexible Weak PolyElectrolyte
HH	Henderson–Hasselbalch
IDP	Intrinsically Disordered Protein
LEIP	Local Effective Interaction Parameters
LPEI	Linear PolyEthileneImine
LR	Long Range
MC	Monte Carlo
MCr	Macromolecular Crowding
MD	Molecular Dynamics
MM	Molecular Mechanics
NMR	Nuclear Magnetic Resonance
PAA	PolyAcrylic Acid
PE(s)	PolyElectrolyte(s)
PMAA	Poly(MethAcrylic Acid)
QM	Quantum Mechanics
RIS	Rotational Isomeric State
SB	Site Binding
SBRIS	Site Binding Rotational Isomeric State
SR	Short Range
TM	Transfer Matrix
WSIP	Wrong Side of the Isoelectric Point
WLC	Worm-Like Chain
